# Integrated transcriptomic and metabolomic analyses revealed the regulatory mechanism of sulfur application in grain yield and protein content in wheat (*Triticum aestivum* L.)

**DOI:** 10.3389/fpls.2022.935516

**Published:** 2022-09-16

**Authors:** Zhilian Liu, Dongcheng Liu, Xiaoyi Fu, Xiong Du, Yuechen Zhang, Wenchao Zhen, Shan Li, Haichuan Yang, Suqin He, Ruiqi Li

**Affiliations:** ^1^State Key Laboratory of North China Crop Improvement and Regulation, College of Agronomy, Hebei Agricultural University, Baoding, China; ^2^Wheat Breeding Center, Gaocheng Institute of Agricultural Sciences, Shijiazhuang, China; ^3^Wheat Research Center, Shijiazhuang Academy of Agriculture and Forestry Sciences, Shijiazhuang, China; ^4^Agricultural Technology Promotion Center, Gaocheng Agricultural and Rural Bureau of Shijiazhuang City, Shijiazhuang, China

**Keywords:** bread wheat, sulfur, transcriptome, metabolome, kernel weight, protein content

## Abstract

Sulfur fertilizers play an important role in increasing the yield and improving the dough quality of bread wheat, but their regulatory mechanism remains unclear. In this study, 0 kg·ha^−1^ (S0) and 60 kg·ha^−1^ (S60) of sulfur were applied on the anthesis date; subsequently, immature wheat grains at 8, 13, and 18 days post-anthesis (DPA) were subjected to integrated transcriptomic and metabolomic analyses to investigate the changes in the gene/metabolite activity in a typical strong-gluten wheat, Gaoyou2018 (GY2018). Our data show that the S60 treatment could significantly increase the grain yield and grain protein content by 13.2 and 3.6%, respectively. The transcriptomic analysis revealed that 10,694 differentially expressed genes (DEGs) were induced by S60 from 8 to 18 DPA when compared with their corresponding no-sulfur controls, and most DEGs were mainly involved in lipid metabolism and amino acid metabolism pathways. Ninety-seven MYB transcription factors (TFs) were identified as responsive to the S60 treatment; of these, 66 showed significantly differential expression at 13 DPA, and MYB118 might participate in the process of sulfur metabolism by regulating glucosinolate synthesis. In total, 542 significantly enriched differentially expressed (DE) metabolites (DEMs) were identified following the S60 treatment, which mainly included secondary metabolites, carbohydrates, and amino acids. Several metabolites (e.g., glutathione, sucrose, GDP-alpha-D-glucose, and amino acids) exhibited altered abundances following the S60 treatment. The combination of transcriptomic and metabolomic analyses highlighted the important role of amino acid metabolism (especially cysteine, methionine, and glutathione metabolism) and starch and sucrose metabolism pathways after S60 application. Our results provide valuable information enhancing our understanding of the molecular mechanism of the response to sulfur and provide useful clues for grain protein quality formation and yield improvement in bread wheat.

## Introduction

Bread wheat (*Triticum aestivum* L.) is an important food crop worldwide (Wang et al., 2015) and increasing its yield and improving its quality have always been major breeding targets to meet the dietary needs of the growing population (Yu et al., 2021). However, these two agronomically most important traits always show a negative correlation (Blanco et al., 2012). Alleviating the contradiction between a high yield potential and an elite quality is a challenging task for wheat breeders.

Sulfur is an essential macronutrient for wheat (Fuentes-Lara et al., 2019; Li et al., 2020). Under sufficient sulfur fertilization, the grain yield increased 3-fold, and the harvest index and nitrogen use efficiency were enhanced by 31.0 and 16.0%, respectively (Zörb et al., 2009; Yu et al., 2018), whereas sulfur deficiency severely affected the photosynthetic apparatus, inhibited the photosynthetic efficiency, and decreased the final grain dry mass and yield (Resurreccion et al., 2002; Dai et al., 2015; Bouranis et al., 2020). Simultaneously, sulfur played a vital role in grain protein synthesis, especially the dough quality-related proteins glutenin and gliadin (Shewry and Halford, 2002; Veraverbeke and Delcour, 2002; Delcour et al., 2012; Theethira and Dennis, 2015). Compared to 0 kg·ha^−1^, the application of sulfur at 50 kg·ha^−1^ (soil) resulted in a sharp increase in the protein content from 18.3 to 20.6% (Yu et al., 2018), and the glutenin to gliadin ratio, an important parameter of the elite dough quality, was almost doubled under sulfur application of 30 kg·ha^−1^ (Yu et al., 2021). Sulfur is also a constituent of amino acids, chloroplasts, sulfatides, and vitamins (Nakai and Maruyama-Nakashita, 2020). Its deficiency not only led to low metabolic activities (Bielecka et al., 2014; Forieri et al., 2017) but also reduced the proportion of sulfur-containing amino acids, such as cysteine and methionine, resulting in a blockage of protein biosynthesis (Li et al., 2020). Moreover, sulfur deficiency induced the accumulation of free aspartic acid, which tended to promote the formation of acrylamide and other potentially hazardous compounds during the bread-making process (Granvogl et al., 2007). Thus, the appropriate sulfur application could be beneficial for increasing the grain yield and synthesis of sulfur-containing proteins for improving the grain's nutritional value (Curtis et al., 2009; Zörb et al., 2009).

Plants adopt strategies to adjust their metabolism and maintain proper development in response to nutrient imbalance (Bonnot et al., 2020). To understand the sulfur metabolic mechanism, genomic, transcriptomic, and metabolomic investigations and physiological and molecular regulatory studies have been widely performed (Watanabe and Hoefgen, 2019; Aarabi et al., 2020). In Arabidopsis, the synthesis of glucosinolate, a sulfur-rich secondary compound in *Brassicaceae*, was inhibited under sulfate starvation conditions *via* the downregulated expression of MYB29, a positive regulatory factor responsible for aliphatic glucosinolate biosynthesis (Gigolashvili et al., 2008), and this inhibition was recovered by the derepressed expression of MYB29 upon sulfate resupply (Bielecka et al., 2014). In bread wheat, sulfur metabolism was significantly affected under sulfur deficiency treatment, and the genes related to the synthesis of grain storage and non-prolamin proteins were predominantly downregulated, resulting in a decreased accumulation of corresponding storage proteins (Dai et al., 2015). However, sulfur supply strongly increased the rate of accumulation of sulfur-rich α/β-gliadin and γ-gliadin and proteins involved in glutathione metabolism in einkorn wheat (Bonnot et al., 2020). In soybean, the transcriptional activation of cysteine, methionine, and glutathione metabolism pathways induced the accumulation of sulfur-containing amino acids (cysteine and methionine) in low-β subunit content seeds, suggesting that sulfur assimilation is involved in β subunit accumulation (Zhang et al., 2019). These data demonstrate that sulfur could balance proteins by regulating the accumulation of sulfur-containing amino acids or metabolites in seed/seedling development.

Sulfur plays a vital role in grain yield and protein synthesis. However, its regulatory mechanism is still unclear. In this study, to understand the effects of sulfur on protein biosynthesis and grain yield formation, a high sulfur treatment was applied to a strong-gluten wheat variety, GY2018. Integrated transcriptomic and metabolomic analyses were conducted in developing kernels. The key DEGs involved in grain quality and the starch formation process were identified. These DEGs might be important regulators of grain yield and dough quality in relation to sulfur metabolism.

## Materials and methods

### Plant material and field trials

A typical strong-gluten wheat variety, GY2018, was used as the plant material; GY2018 was released in Hebei Province, China, in 2008 and widely planted in the middle and southern parts of Hebei Province. In this experiment, GY2018 was sown in vermiculite-filled pots in Gaocheng Institute of Agricultural Sciences, China (114.84° E 38.03° N) on 26 October 2019, and harvested on 9 June 2020, and the pots were placed in a natural environment under the same conditions as field production (Supplementary Figure S1). Two sulfur treatments, 0 kg·ha^−1^ and 60 kg·ha^−1^ of sulfur application, were performed as a no-sulfur control (S0) and high-sulfur treatment (S60), respectively. Potassium sulfate was utilized as the source of sulfur, and for the S0 treatment, potassium chloride was applied accordingly. Commercial urea (46% nitrogen), calcium superphosphate (P_2_O_5_ ≥ 46%) and potassium chloride (K_2_O ≥ 60%) were fertilized as nitrogen (N), phosphorus(P), and potassium (K) sources at 240 kg·ha^−1^, 120 kg·ha^−1^, and 177 kg·ha^−1^, respectively. The total amount of P and K and half of N were applied as the basal dose before sowing, and the remaining 50% of N was topdressed during the jointing stage. The micronutrient fertilizers were supplemented with a modified Hoagland nutrient solution (Castle and Randall, 1987). The size of the polyethylene pot was 24 cm × 24 cm × 27 cm, 40 seeds were sown and thinned to 15 seedlings after emergence in each pot, and each treatment was repeated three times. Sulfur was applied on the anthesis date. Following the S60 and S0 treatments, developing grains at 8, 13, and 18 days post-anthesis (DPA) were collected, resulting in six tissues for transcriptome and metabolome profiling. From each pot, the central grains of each spike were collected from the main spikes, frozen immediately in liquid nitrogen, and then stored at −80°C for RNA and metabolite extraction. Three and six biological replicates were performed per time point, resulting in 18 and 36 samples for the transcriptomic and metabolomic analyses, respectively. From each pot, 10 main tillers from 10 individual plants were harvested upon maturation. The grain weight and biomass were measured and averaged per culm to represent the yield performance under the sulfur treatments.

### Measurement of plant dry matter and grain protein content

At the fully mature stage, the above-harvested tillers per pot were separated into the following four components by hand: grains, glumes, leaves, and stems. Air-dried mature grains were used to calculate the thousand kernel weight (TKW), and each component was dried for 48 h at 70°C for the measurement of the dry weight. The dried grains were pulverized (<0.20 mm) and subjected to a total nitrogen content measurement using an automatic nitrogen analyzer (AutoAnalyzer, AA3, SEAL). The grain protein content (GPC) was calculated as the grain nitrogen content multiplied by 5.7 (Nehe et al., 2020). The amino acid content was measured with an amino acid content kit (Solarbio, Beijing, China) (Li et al., 2017). Both the GPC and amino acid content were measured with three biological replications.

### RNA extraction and sequencing

The total RNA was extracted from immature grains with a NEBNext^®^ UltraTM RNA Library Prep Kit for Illumina^®^ (NEB, USA), and index codes were added to attribute sequences to each sample. Briefly, mRNA was purified from total RNA using poly-T oligo-attached magnetic beads. Fragmentation of mRNA was carried out using divalent cations under an elevated temperature in the NEBNext First Strand Synthesis Reaction Buffer. The prepared libraries were sequenced on an Illumina NovaSeq platform, 150 bp paired-end reads were generated, and more than 82,325,260 bp reads were obtained per sample. Adaptor sequences and low-quality sequence reads were removed from the raw datasets, and the resultant clean data were retained with their Q20 and GC content for further downstream analyses. The clean reads were aligned to the wheat reference genome sequences of Chinese Spring (ver. 2.0), (ftp://ftp.ensemblgenomes.org/pub/release46/plants/fasta/triticum_aestivum/dna/Triticum_aestivum.IWGSC.dna.toplevel.fa.gz) released by the International Wheat Genome Sequencing Consortium (IWGSC). The index of the reference genome was built using HISAT2 (v2.0.5) and paired-end clean reads were aligned to the reference genome. The sample identity was designated based on the RNA sequencing data (Supplementary Table S1).

### Quantification of gene expression and differential expression analysis

FeatureCounts (v1.5.0-p3), which is available under the GNU General Public License as a part of Subread (http://subread.sourceforge.net), was used to count the reads mapped to each gene (Liao et al., 2014). The expression levels of each gene were calculated and normalized by the corresponding fragments per kilobase of transcript per million mapped fragments (FPKM). The average FPKM across the three biological replicates was calculated for each sample and subjected to a DEG analysis. A differential expression analysis of two conditions/groups (S60 vs. S0) was performed. The DEGs were identified based on a negative binomial distribution model with the DESeq 2 R package (v 1.16.1) (Love et al., 2014). An FPKM value >0.02 in at least one of the three time points of grain development was considered effective gene expression. The *P-*values were adjusted using the Benjamini & Hochberg approach to control for the false discovery rate. A corrected *P-*value of 0.05 (padj < 0.05) and an absolute logarithm 2 fold change (|log_2_ (fold change)| ≥ 1) were set as the thresholds for significant DEGs. The clusterProfiler R package (v 3.4.4) was employed to test the statistical enrichment of the differentially expressed genes in the Kyoto Encyclopedia of Genes and Genomes (KEGG) pathway and the Gene Ontology (GO) enrichment analyses. Statistically significant overrepresentation of GO categories (padj < 0.05) in response to the sulfur treatments was determined separately in each grain developmental stage.

### Untargeted metabolomics analysis

Metabolite profiling was carried out using a widely untargeted metabolome method by Beijing Novogene Biotechnology Co., Ltd. (Beijing, China) (http://www.novogene.com/). Metabolites were extracted following an available protocol (Want et al., 2013), and untargeted metabolites were screened by ultrahigh-performance liquid chromatography-tandem mass spectrometry (UHPLC–MS/MS). The UHPLC–MS/MS analyses were performed using a Vanquish UHPLC system (Thermo Fisher, Germany) coupled with an Orbitrap Q Exactive™ HF mass spectrometer (Thermo Fisher, Germany) by Novogene Co., Ltd. (Beijing, China). The samples were injected onto a Hypesil Gold column (100 mm × 2.1 mm, 1.9 μm) using a 17-min linear gradient at a flow rate of 0.2 mL/min. The eluents for the positive polarity mode were eluent A (0.1% FA in water) and eluent B (methanol), and the eluents for the negative polarity mode were eluent A (5 mM ammonium acetate, pH 9.0) and eluent B (methanol). The solvent gradient was set as follows: 2% methanol, 1.5 min; 2–100% methanol, 12.0 min; 100% methanol, 14.0 min; 100–2% methanol, 14.1 min; and 2% methanol, 17 min. The Q Exactive™ HF mass spectrometer was operated in the positive/negative polarity mode with a spray voltage of 3.2 kV, a capillary temperature of 320°C, a sheath gas flow rate of 40 arb, and an aux gas flow rate of 10 arb. The data preprocessing steps included sample standardization, metabolite dereplication, and setting the threshold standard of the coefficient of variation (CV) as follows: sample standardization: standardized treatment of quantitative values by untargeted metabolism; formula: the original quantitative value of the sample metabolite/(sum of the quantitative value of the sample metabolite/sum of the quantitative value of the quality control (QC)1 sample metabolite); metabolite dereplication: performed according to the database priority- mzCloud > mzVault > MassList; and CV threshold standard: metabolites with a CV <0.3 in the QC samples were retained, and metabolites with a CV >0.3 in the QC samples were excluded. After extraction from the chromatograms, the data were processed, aligned, and filtered using the Compound Discoverer software (v 3.1). The normalized data were used to predict the molecular formula based on additive ions, molecular ion peaks, and fragment ions. The metabolite structures were analyzed with the mzCloud (https://www.mzcloud.org/), mzVault, and MassList databases to obtain accurate qualitative and relative quantitative data. The statistical analyses were performed using the software R (v 3.4.3), Python (v 2.7.6) and CentOS (v 6.6). All metabolites were annotated with the KEGG database (https://www.genome.jp/kegg/pathway.html). A principal component analysis and a partial least squares discriminant analysis were performed at metaX (Wen et al., 2017). The statistical significance was calculated with a *t-*test. Metabolites with VIP > 1, *P*-value ≤ 0.05 and fold change >1.5 or <0.67 between the two treatments (S60 vs. S0) were considered DEMs.

### Quantitative reverse transcription PCR (qRT–PCR) validation

To validate the RNA-seq results, 12 DEGs were selected for a quantitative reverse transcription PCR (qRT–PCR) analysis. Based on the mRNA sequences obtained from the National Center for Biotechnology Information (NCBI) database, primers were designed with Primer 5 software (v 5.0) and synthesized by TsingKe Biotech (Supplementary Table S2). Quantification was performed with the following two-step reaction process: reverse transcription (RT) and PCR. Each RT reaction consisted of 0.5 μg RNA, 2 μl of 5× TransScript All-in-one SuperMix for qPCR and 0.5 μl of gDNA Remover in a total volume of 10 μl. The reactions were performed on a GeneAmp^®^ PCR System 9700 (Applied Biosystems, USA) for 15 min at 42°C and 5 s at 85°C. The resultant RT solution was diluted to 100 μl, and 2 μl was used for each qRT–PCR analysis. qRT–PCR was performed using a LightCycler^®^ 480 II Real-time PCR Instrument (Roche, Switzerland) with a 10 μl PCR mixture. The reactions were incubated in a 384-well optical plate (Roche, Switzerland) at 94°C for 30 s, followed by 45 cycles of 94°C for 5 s and 60°C for 30 s. Three replicates of each sample were performed. The normalized relative quantities relative to the reference gene (Radonić et al., 2004) were calculated using the 2^−ΔΔCt^ method (Livak and Schmittgen, 2001).

### Integration analysis of the transcriptome and metabolome

All DEGs and DEMs were simultaneously mapped to the KEGG pathway database to obtain their common pathway information and determine the main biochemical pathways and signal transduction pathways. We used the clusterProfiler R package (R-3.4.3) and Python (python3.5.0) to test the common statistical enrichment KEGG pathways of the DEGs and DEMs. The joint analysis was carried out using the metabolome and transcriptome data with the standard of a *P-*value ≤ 0.05.

### Statistical analysis

A one-way analysis of variance (ANOVA) was performed using SPSS 2019 Software (SPSS Inc. Chicago, IL, USA). The means of two samples were compared using Student's two-tailed *t-*tests.

## Results

### Sulfur application significantly improved wheat yield

**Figure 1:**
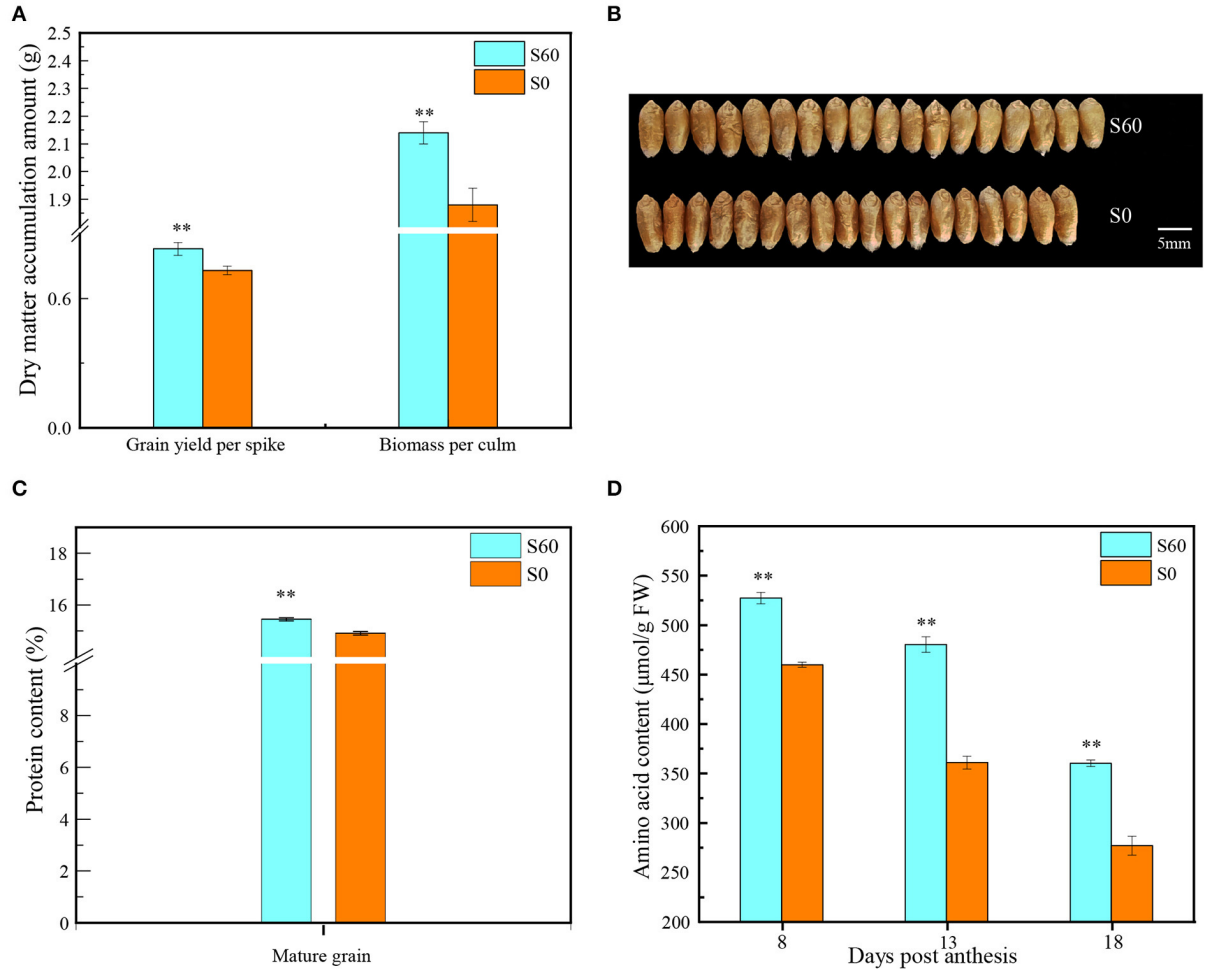
Yield, protein content, and amino acid indices in the wheat variety GY2018. **(A)** Grain yield and biomass at maturity (data show the content of a single culm) (*t*-*test, P* < 0.01). **(B)** Photograph of grains at the mature stage, top row, grains under the S60 treatment; bottom row, grains under the S0 treatment. The light blue column represents the high-sulfur treatment (S60), and the orange column represents the no-sulfur treatment (S0). **(C)** Grain protein concentration (GPC) in mature grains (*t*-*test, P* < 0.01). **(D)** Dynamic changes in the amino acid level at different grain developmental stages at 8, 13, and 18 DPA (*t-test, P* <0.01). Error bars represent the SD of three replicates. DPA, days post-anthesis. **P < 0.01.

In comparison with S0 (no-sulfur application), the biomass and grain yield following the S60 treatment (60 kg·ha^−1^ sulfur treatment) increased from 1.9 to 2.1 g per culm and 0.7 to 0.8 g per culm (*P* < 0.001), with a positive increase of 13.9 and 13.2%, respectively (*P* < 0.01) (Figure 1A). The grain plumpness following the S60 treatment was better than that following the S0 treatment, and the resultant TKW significantly increased from 44.9 g following the S0 treatment to 46.5 g following the S60 treatment (*P* < 0.001) (Figure 1B). As expected, the GPC was simultaneously enhanced from 14.9% following the S0 treatment to 15.5% following the S60 treatment (*P* < 0.001) (Figure 1C). To detect the change in GPC, the total amino acids of developing grains were measured. At 8 DPA, the amino acids increased from 459.9 μmol/g FW following the S0 treatment to 527.3 μmol/g FW following the S60 treatment, resulting in a 14.7% increase, and this increase reached 33.1 and 30.1% at 13 and 18 DPA, respectively (*P* < 0.01) (Figure 1D). Moreover, along with grain development, the total amino acids decreased from 459.9 μmol/g FW at 8 DPA to 361.0 μmol/g FW at 13 DPA and then to 277.0 μmol/g FW at 18 DPA (S0), and there was a similar trend in the S60 plants, suggesting that free amino acids were consumed for protein synthesis in later grain developmental stages. Thus, the biomass, grain yield, TKW, and GPC were significantly enhanced by the sulfur application, demonstrating that sulfur fertilization could have beneficial effects on the yield potential and protein content in strong-gluten wheat.

### Transcriptional response to sulfur treatments in GY2018

**Figure 2:**
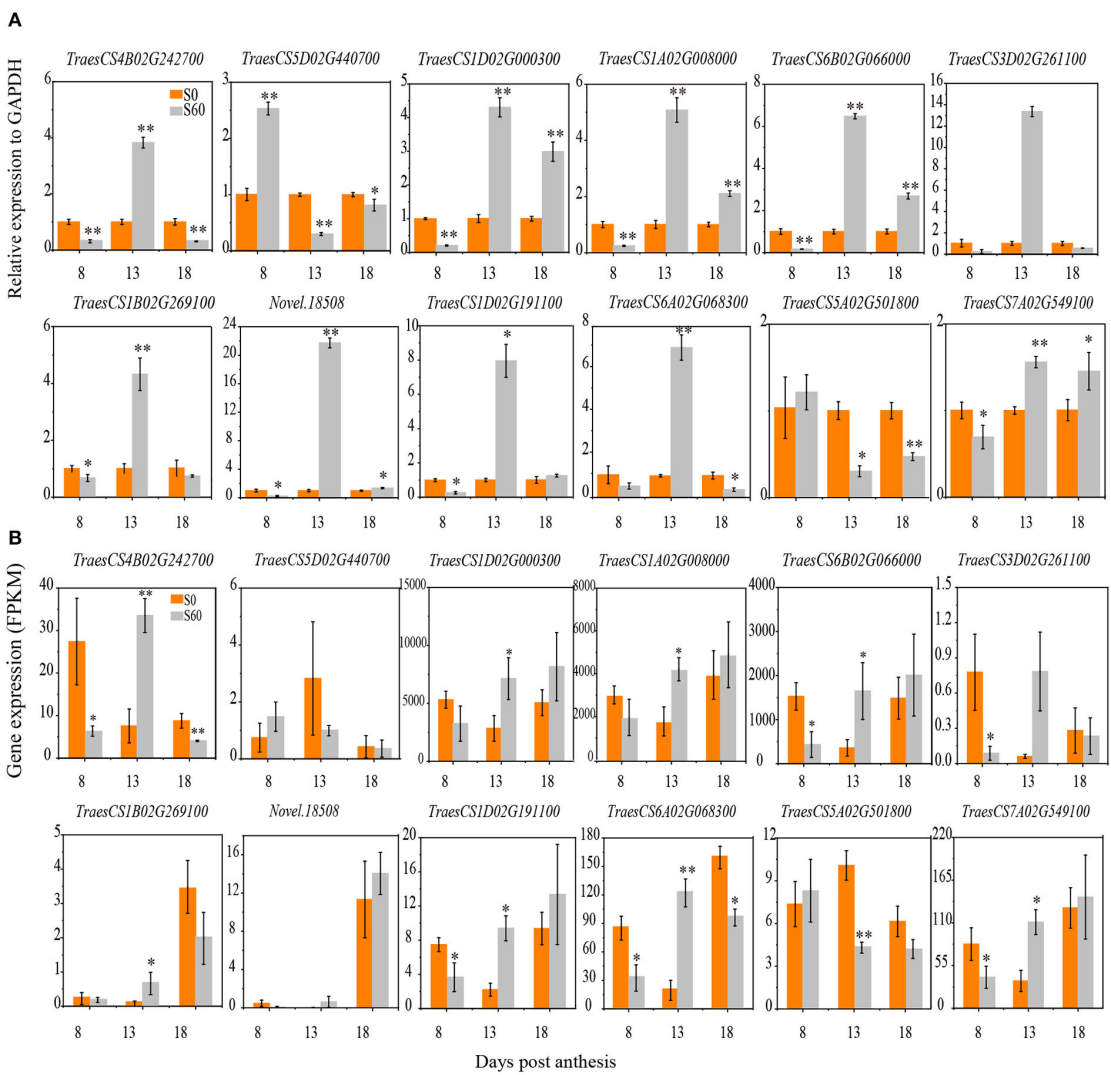
Expression profiles of gene validation under the S60 and S0 treatments using a qRT–PCR analysis. Twelve participating DEGs were selected for qRT–PCR. The relative levels of gene expression were calculated against GAPDH as the reference gene. The x-axis shows the 8, 13, and 18 DPA samples at grain developmental stages. The orange column represents the high-sulfur treatment (S60), and the gray column represents the no-sulfur treatment (S0). **(A)** The y-axis represents the expression profiles of five DEGs in the qRT–PCR analysis. **(B)** The y-axis represents the expression profiles of five DEGs shown by the fragments per kb per million reads (FPKM) values in the RNA-Seq analysis. DEGs, differentially expressed genes; DPA, days post-anthesis. All data are presented as the mean ± standard deviation of three biological replicates. *P < 0.05; **P < 0.01.

The RNA-seq analysis of 18 samples yielded more than 157 million clean reads with a GC content of ~53%. The proportion of clean reads mapped to a unique location of the wheat reference genome ranged from 79.3 to 87.5% (Supplementary Tables S3, S4). In total, 140,829 transcripts were obtained; of these, 20,085 were novel transcripts (Supplementary Table S5). To investigate the reproducibility of the biological replicates and the relationship among the different samples, a correlation analysis was performed based on the global gene expression pattern. Pearson's correlation analysis was performed to confirm the reproducibility between the biological replicates, and the resultant *R*^2^ was over 0.90 (Supplementary Figure S2). Thus, the FPKM values of three biological replicates of each tissue were averaged to represent their expression abundance in each treatment and developmental stage. Furthermore, the qRT–PCR data of 12 randomly selected genes were highly correlated (*R*^2^ = 0.85) with those obtained from the RNA-seq analysis (Figures 2A,B, Supplementary Figure S3), confirming the accuracy of the transcriptome data obtained by RNA-seq.

**Figure 3:**
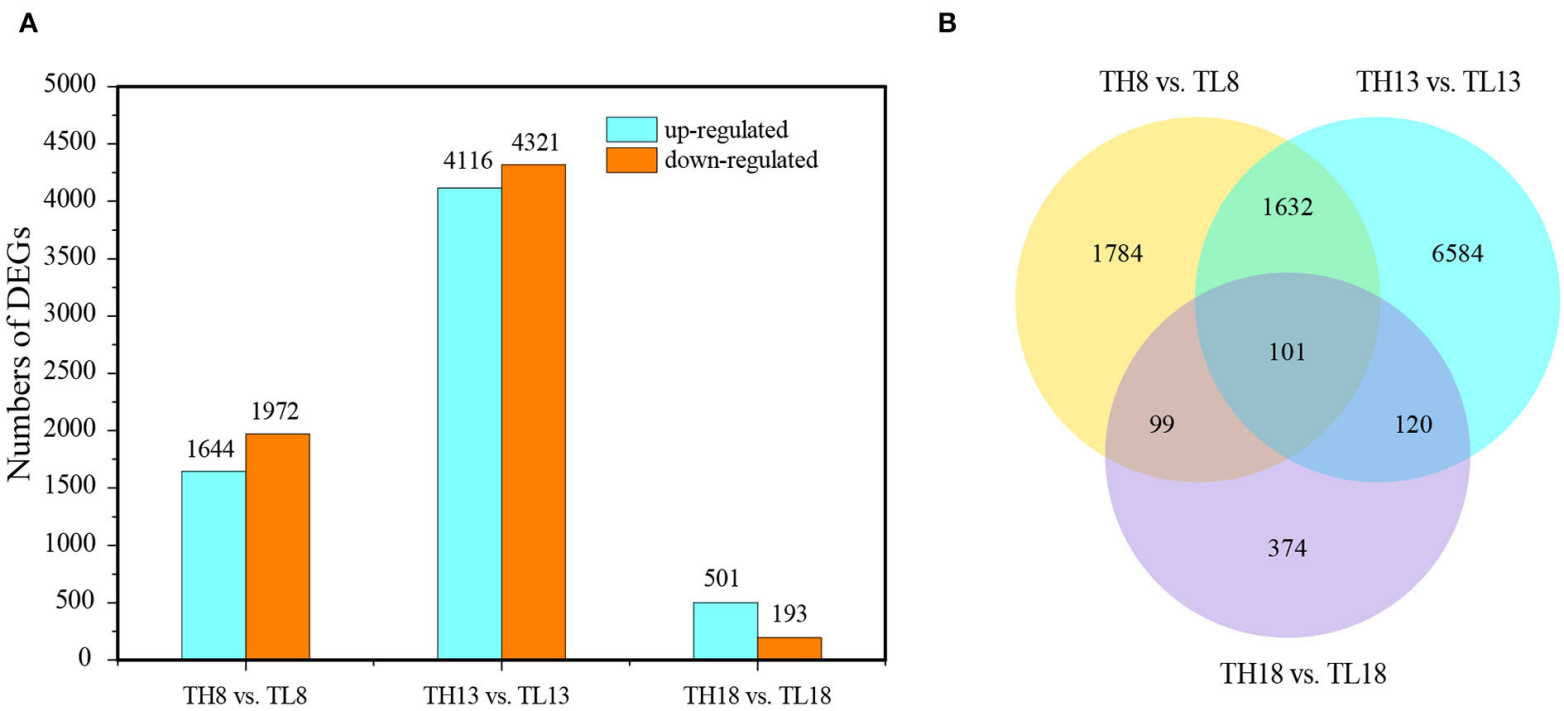
Overview of the transcriptome analysis of the TH8 vs. TL8, TH13 vs. TL13, and TH18 vs. TL18 comparison groups of GY2018. **(A)** Number of upregulated and downregulated DEGs between the S60 and S0 combination groups. The light blue column represents the transcriptome levels of the upregulated DEGs, and the orange column represents the downregulated DEGs. **(B)** Venn diagram of DEGs under the S60 and S0 combination. DEGs, differentially expressed genes; DPA, days post-anthesis.

**Figure 4:**
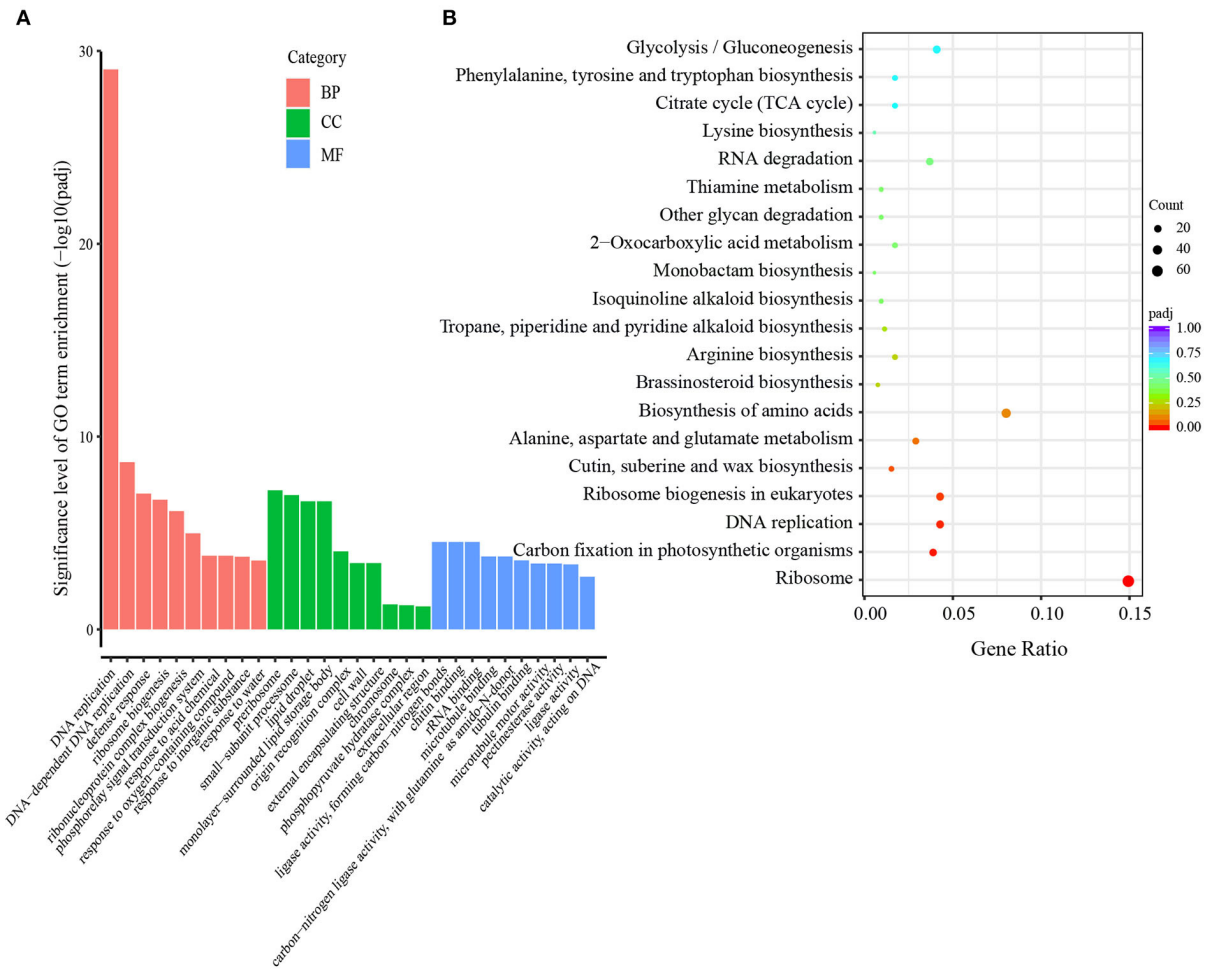
GO classification and KEGG enrichment of DEGs in the TH13 vs. TL13 comparison groups of GY2018. **(A)** Significantly enriched GO terms (corrected *P-*value ≤ 0.05). The y-axis represents the significance level of GO term enrichment, and the x-axis represents each GO term. Biological process, red bar; Cellular component, green bar; Molecular function, blue bar. **(B)** KEGG pathway enrichment scatters diagram of the DEGs in filling grain of GY2018 at 13 DPA. The colors of the dots indicate the correlated *P-*value, and the sizes of the dots indicate the input number. DPA, days post-anthesis.

The DEGs between the two treatments were analyzed at each grain developmental stage. At 8 DPA, the early grain developmental stage of cellularization and differentiation, in total, 3,616 DEGs were detected between the S60 (TH8) and S0 (TL8) treatments; of these, 1,644 were upregulated, and 1,972 were downregulated (Figures 3A,B). Through a GO enrichment analysis, these DEGs were enriched in terms associated with cell movement (e.g., motor activity and cytoskeletal protein binding) and stress-responsive activities (e.g., response to water and defense response) (Supplementary Figures S4A,B). Hence, at the early grain developmental stage, sulfur might promote cell propagation and expansion. At the early grain filling stage (13 DPA), the high sulfur treatment TH13 exhibited 4,116 upregulated and 4,321 downregulated DEGs compared to no- sulfur supply (Figures 3A,B). The significantly enriched GO terms were protein synthesis, the formation of carbon and nitrogen skeletons (e.g., DNA replication, carbon-nitrogen ligase activity, and glutamine as amido-N-donor), and plant defense (e.g., chitin binding, defense response, and chitinase activity; Figure 4A). These DEGs were mainly enriched in lipid metabolism and amino acid metabolism through the KEGG pathway enrichment analysis. This process could provide more substrate sources for protein synthesis in developing grains, suggesting that sulfur application might activate the development of liposomes and proteosomes (Figure 4B). With the extension of the grain filling process, endosperm cells stop dividing, and protein and starch granules accumulate largely at the later grain filling stage (Laudencia-Chingcuanco et al., 2007; Zhang et al., 2021). Only 694 DEGs were identified between the high-sulfur-treatment TH18 and no-sulfur supply TL18; of these, 501 and 193 were upregulated and downregulated, respectively. Most GO terms belonged to the biological process category (Supplementary Figure S5A). At this stage, only protein processing in the endoplasmic reticulum pathway was significantly enriched (Supplementary Figure S5B), suggesting that the involvement of sulfur can promote the rapid accumulation of grain protein. The Venn diagram analysis showed that the three groups shared 101 DEGs. The GO terms were mainly involved in grain development (e.g., embryo development and developmental process; Supplementary Figure S6A) and plant response (e.g., response to abiotic stimulus and defense response). Protein processing in the endoplasmic reticulum and the cysteine and methionine metabolism pathways were significantly enriched (Supplementary Figure S6B). Furthermore, most of the 101 DEGs were downregulated (87 DEGs, accounting for 86.1%) at 8 DPA, while 66 genes were upregulated, and 11 genes were downregulated at 13 DPA and 18 DPA between the S60 and S0 treatments (Supplementary Table S6).

**Figure 5:**
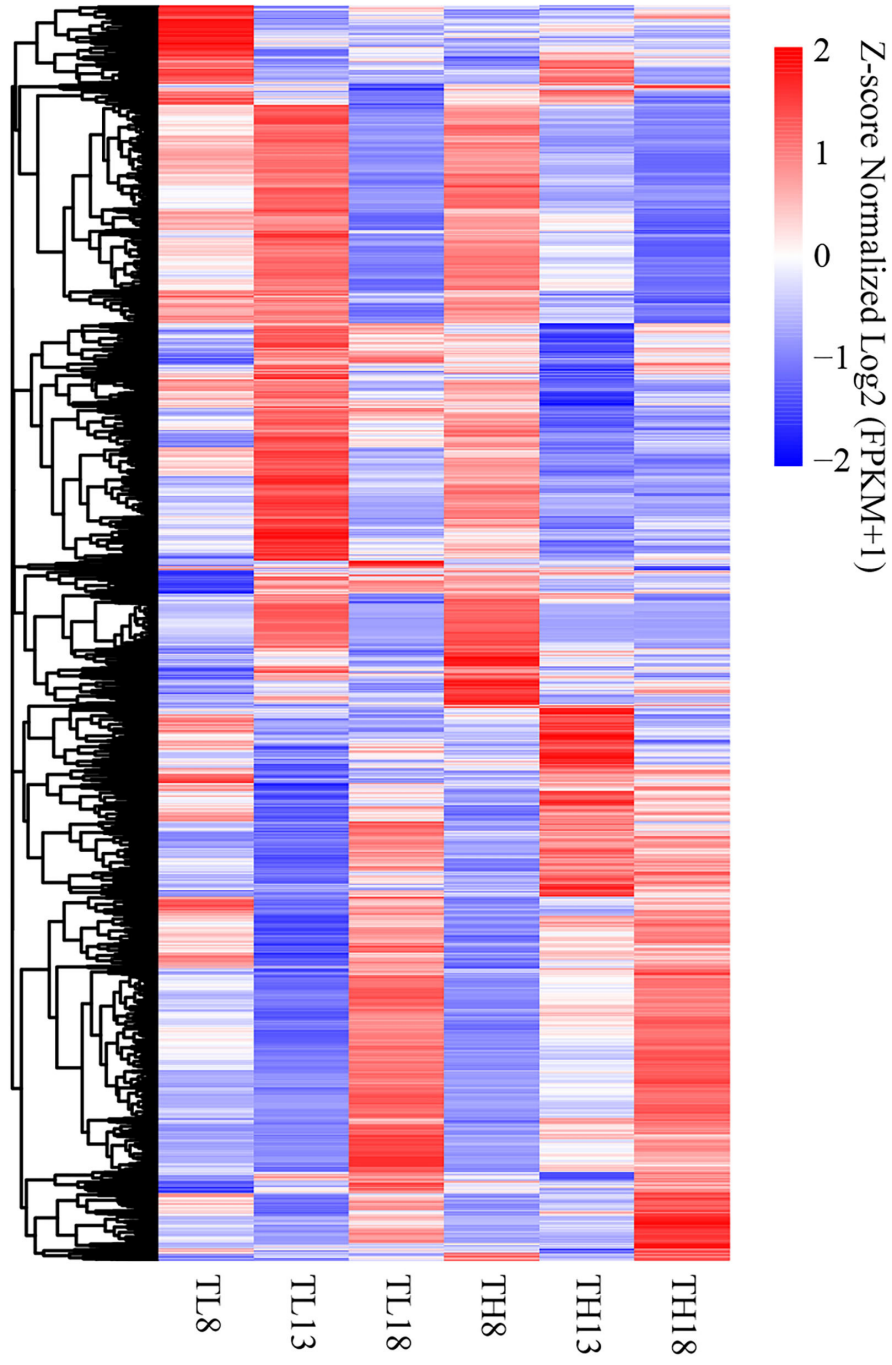
Heatmap of the accumulation patterns of the 10,694 DEGs in six tissues. Log2 (FPKM+1) was extracted from the expression quantity of the DEGs by the hierarchical clustering method. The labels are TL8 (8 DPA, S0), TH8 (8 DPA, S60), TL13 (13 DPA, S0), TH13 (13 DPA, S60), TL18 (18 DPA, S0), and TH18 (18 DPA, S60). The same label in the following figures has the same meaning. The level of normalized gene expression from high to low is indicated by the color scheme from red to white to blue. DPA, days post-anthesis.

At the above three key grain developing stages, in total, 10,694 DEGs were detected accumulatively between the two sulfur treatments, and hierarchical clustering was performed (Figure 5, Supplementary Table S7). The sulfur application had the most significant effect on DEG expression in developing grains at 13 DPA, which was also the period of the formation of grain morphology and the development of endosperm cells. At 8 DPA, the expression patterns of the DEGs differed from those at 13 DPA, while the DEGs at 18 DPA had similar accumulation profiles between the two sulfur treatments. These results highlight the strong effect of grain development and sulfur application on the transcriptome.

### Transcription factor analysis of the GY2018 response to sulfur treatments

TFs are important regulators of plant responses to sulfur treatments. Based on the transcriptome data, 374 DE TFs were identified (Supplementary Table S7); among these, the most significant members (>70) were Pkinase, Histone, p450, AP2, and Myb_DNA-binding (Supplementary Table S8). In total, 66 DE MYB TFs were observed under the sulfur treatments at 13 DPA; of these, 32 were upregulated and 34 were downregulated. These MYBs (Supplementary Table S9) are potentially involved in plant growth and development and stress (e.g., lignin biosynthesis, drought stress, morphogenesis regulation, fruit development regulation, and cell cycle regulation) (Fujiwara et al., 2014; Geng et al., 2020; Gao X. et al., 2021; Li D. et al., 2021; Takatsuka et al., 2021). Among them, *TraesCS3B02G400500* had a high similarity to *AtMYB118*, which mainly regulates glucosinolate biosynthesis along with *AtMYB115* (Zhang et al., 2015). Therefore, this DEG may play an important role in the process of sulfur metabolism.

### Metabolic characteristics of the GY2018 response to sulfur treatments

**Figure 6:**
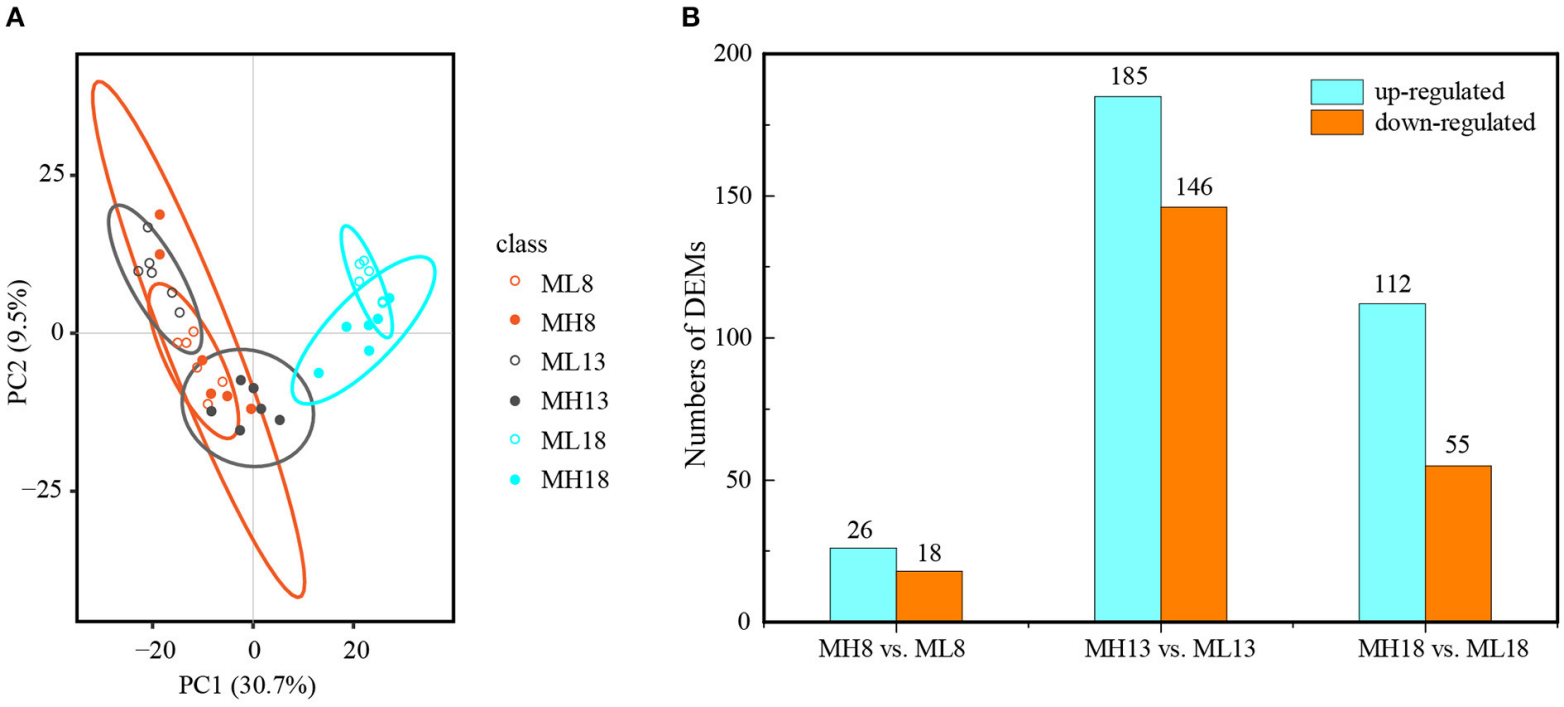
Overview of the metabolome analysis of the MH8 vs. ML8, MH13 vs. ML13, and MH18 vs. ML18 comparison groups of GY2018. **(A)** Principal component analysis of the metabolic profiles of GY2018. **(B)** Bar chart of the DEMs between the S60 and S0 combination groups. The labels are ML8 (8 DPA, S0), MH8 (8 DPA, S60), ML13 (13 DPA, S0), MH13 (13 DPA, S60), ML18 (18 DPA, S0), and MH18 (18 DPA, S60). PC: principal component; the same labels are used in **(A,B)**. The same label in the following figures has the same meaning. Six independent biological replicates were used for the metabolome analysis. DPA, days post-anthesis.

The principal component analysis showed that the developmental stages as the first principal component (PC1) could explain 30.7% of the total variation, while the sulfur treatments were the second principal component (PC2), explaining 9.5% of the variation across the dataset. Thus, most of the variation in metabolites among different quadrants was a consequence of the developmental stages and sulfur treatments (Figure 6A). Similar to the transcriptome, we performed a pairwise analysis (S60 vs. S0) between the two treatments at three-grain developmental stages (Supplementary Figure S7). All three grains developing in response to the sulfur application were well-separated by PC1, indicating that the changes in the metabolite profiles were caused by the sulfur treatments. Sulfur had the greatest effect on metabolites at 13 DPA since metabolites at this period were clearly divided into two groups, indicating that the metabolites of the S0 and S60 grains clearly differed from each other. The metabolites that were highly accumulated under the S60 treatment at 13 DPA included lipids and lipid-like molecules, organic acids and derivatives, organic nitrogen compounds, and nucleosides (Supplementary Tables S10, S11). A partial least squares discriminant analysis is used to establish a model of the relationship between metabolite expression and sample categories to predict sample categories. Our results show that the *R*^2^ and Q^2^ of the model were close to 1, indicating that the model has a good goodness-of-fit and predictive ability (Supplementary Figure S8).

In total, 542 DEMs were identified in 36 samples (Supplementary Tables S10, S11). The KEGG pathway enrichment analysis revealed that these DEMs were mainly enriched (Q-value ≤ 0.05) in several metabolic processes, including amino acid metabolism, carbohydrate metabolism, biosynthesis of secondary metabolites and metabolism of cofactors and vitamins (Supplementary Figures S9A,B). At the early grain developmental stage (8 DPA), in total, 44 DEMs were identified; of these, 26 were upregulated between the high-sulfur treatment (MH8) and the no-sulfur supply (ML8) treatment (Figure 6B). Among them, D-methionine and L-cystine accumulated 1.9- and 1.6-fold under the sulfur treatments, respectively (Table 1), which could improve protein synthesis in the late stages. Most DEMs were observed at 13 DPA; of these, 185 were upregulated and 146 were downregulated under the sulfur treatments (Figure 6B). Some carbohydrate storage and biosynthesis of amino acid metabolites accumulated mostly in this early grain filling stage (13 DPA), such as raffinose, sucrose, glucose, glutathione, rosmarinic acid, D-glutamine, and S-adenosyl L-homocysteine. In the later grain filling stage (18 DPA), some secondary metabolites were activated and displayed an upregulated pattern (e.g., shikimic acid, ferulic acid, ascorbic acid, and arbutin) (Table 1). The grain yield is largely determined by starch accumulation during the grain filling period in cereals (Smidansky et al., 2002). In our study, starch and protein synthesis compounds (e.g., sucrose, glucose, and amino acid metabolites) were detected with a high abundance under the sulfur treatments, indicating that the sulfur application promoted the accumulation of carbohydrates, protein, and starch granules in the grains and resulted in a higher TKW and protein content (Figures 1B,C).

**Table 1:** Selected DEMs identified in the S60 vs. S0 comparison groups.

**Compound**	**MH8 vs. ML8**		**MH13 vs. ML13**		**MH18 vs. ML18**		**KEGG pathway**
	**Fold change**	**VIP**	**Fold change**	**VIP**	**Fold change**	**VIP**	
D-Methionine	1.92	2.43	0.39	1.73	0.70	0.78	ABC transporters
L-Cystine	1.57	2.88	–	–	–	–	Cysteine and methionine metabolism; ABC transporters
Pheophorbide A	0.45	2.31	–	–	–	–	Porphyrin and chlorophyll metabolism; biosynthesis of secondary metabolites
Glutathione	–	–	12.86	1.94	3.66	1.45	Cysteine and methionine metabolism; glutathione metabolism; metabolic pathways; ABC transporters
Rosmarinic acid	–	–	25.84	2.70	–	–	Tyrosine metabolism
Phosphoenolpyruvic acid	–	–	2.08	1.47	–	–	Glycolysis/ gluconeogenesis citrate cycle (TCA cycle);
Arbutin	–	–	2.66	1.55	2.22	2.03	Glycolysis/gluconeogenesis.
Sucrose	–	–	1.51	1.09	–	–	Galactose metabolism; starch and sucrose metabolism; metabolic pathways; ABC transporters
Raffinose	–	–	1.74	1.19	–	–	Galactose metabolism ABC transporters
GDP-alpha-D-glucose	–	–	1.87	1.15	–	–	Starch and sucrose metabolism; amino sugar and nucleotide sugar metabolism; metabolic pathways
D-Proline	–	–	0.18	2.39	–	–	Arginine and proline metabolism; metabolic pathways
S-Adenosyl L-homocysteine	–	–	0.59	1.2	–	–	Cysteine and methionine metabolism; metabolic pathways; biosynthesis of amino acids
D-Glutamine	–	–	0.29	1.60	–	–	Metabolic pathways
Ascorbic acid	–	–	–	–	6.93	2.24	Ascorbate and aldarate metabolism; glutathione metabolism; metabolic pathways; biosynthesis of secondary metabolites
Thiamin	–	–	–	–	4.00	2.38	Thiamine metabolism, metabolic pathways, ABC transporters, sulfur relay system
Ferulic acid	–	–	–	–	2.28	1.64	Phenylpropanoid biosynthesis, metabolic pathways, biosynthesis of secondary metabolites
Shikimic acid	–	–	–	–	1.56	1.97	Phenylalanine, tyrosine, and tryptophan biosynthesis metabolic; pathways biosynthesis of secondary metabolites; biosynthesis of amino acids

“–” indicate the undetected DEMs.

### Integration of the transcriptome and metabolome profiles in methionine and glutathione metabolism

**Figure 7:**
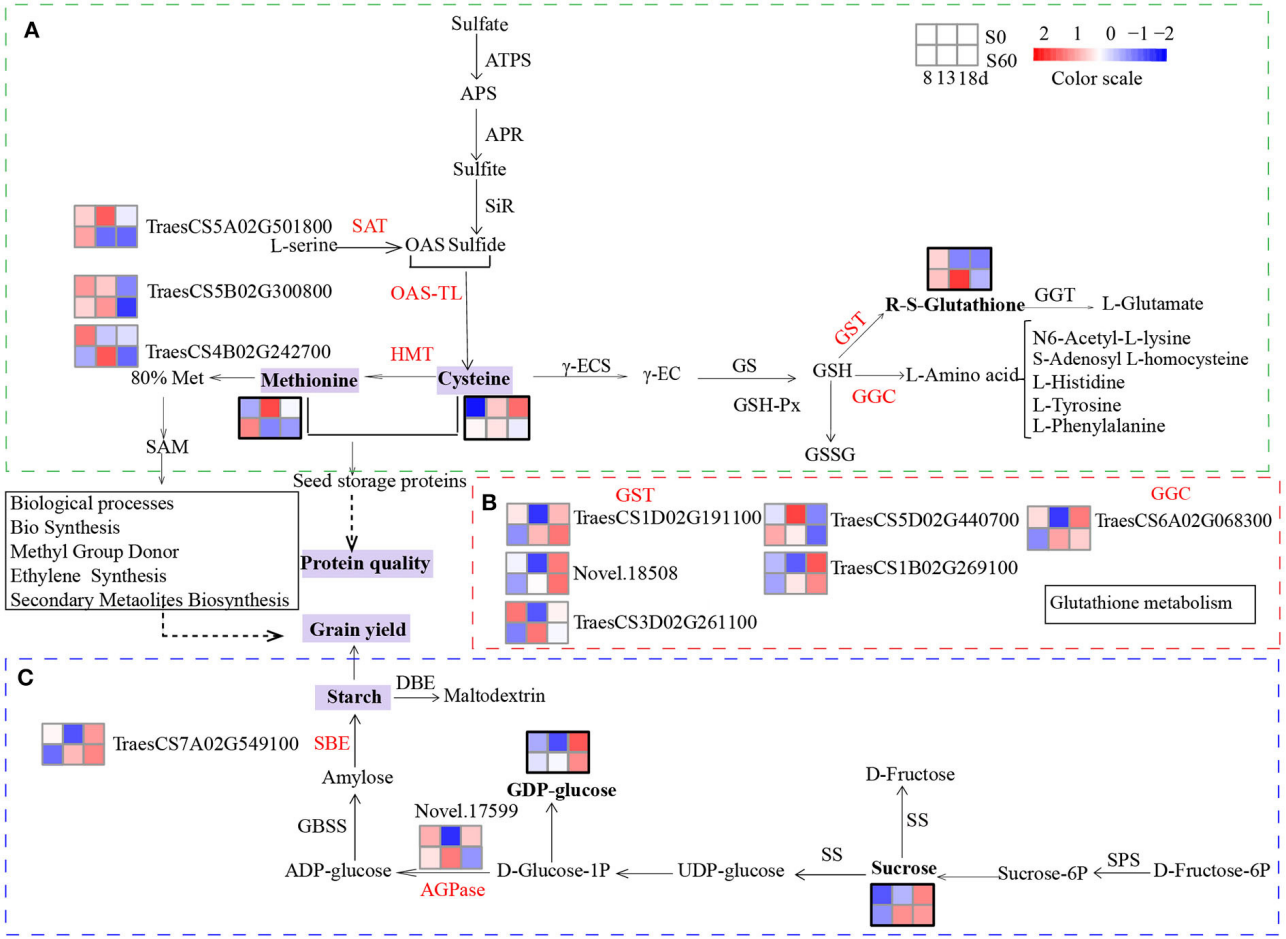
Sulfur-mediated molecular mechanism underlying grain yield and protein quality biosynthesis. Heatmaps showing the effects of sulfur on the DEGs and DEMs by the grain developmental stage. Heatmaps of DEGs: Log2 (FPKM+1) was extracted from the expression quantity of the DEGs by the hierarchical clustering method, and clustering was performed after centralization correction. The red graphic represents the upregulation of gene expression and DEMs, and the blue graphic represents the downregulation of gene expression and DEMs. The heatmaps of the DEMs were calculated by the Z score. DEMs are shown in bold font with a thick black border. Each row represents different sulfur treatments, the top row, grains under the S0 treatment; the bottom row, grains under the S60 treatment. Each column represents different grain development stages at 8, 13, and 18 DPA. **(A)** Synthesis and degradation of cysteine, methionine, and glutathione *via* glutamate and cysteine. **(B)** Heatmaps of the relative expression levels of GST and GGC. **(C)** Synthesis and degradation of sucrose in the starch and sucrose metabolism pathways. ATPS, ATP-sulfurylase; APR, APS reductase; SiR, sulfite reductase; OAS, O-acetyl serine; OAS-TL, O-acetylserine (thiol) lyase; SAT, serine acetyltransferase; HMT, homocysteine S-methyltransferase; γ-ECS, glutamate—cysteine ligase; GS, glutathione synthase; GST, glutathione S-transferase; GSH-Px, glutathione peroxidase; GGC, γ-glutamylcyclotransferase; GGT, γ-glutamyltranspeptidase (glutathione hydrolase); SPS, sucrose-phosphate synthase; SS, sucrose synthase; GBSS, granule-bound starch synthase; SBE, starch branching enzyme; AGPase, ADP-glucose pyrophosphorylase.

When the transcriptome and metabolome profiles were integrated, several common enriched pathways were observed, including cysteine and methionine metabolism and glutathione metabolism (Supplementary Figure S10). The levels of D-methionine were 1.9- and 0.4-fold in the MH8 vs. ML8 and MH13 vs. ML13 comparison groups, respectively (Table 1). Increased methionine synthesis was induced by serine acetyltransferase (SAT), O-acetylserine(thio)lyase (OAS-TL, also called cysteine synthase), and methionine synthase (also called homocysteine S-methyltransferase genes-HMT) mainly through the cysteine and methionine pathways. The analysis of the RNA-seq data indicated that the expression level of a DEG annotated as SAT (*TraesCS5A02G501800*) was significantly downregulated in TH13 vs. TL13. Furthermore, a DEG (*TraesCS5B02G300800*) encoding OAS-TL, which catalyzes O-acetyl-L-serine and sulfide to synthesize cysteine, was also downregulated compared to the no sulfur supply group (TL8). There was a significant increasing trend in the expression of methionine synthase (*TraesCS4B02G242700*, 2.2-fold, TH13 vs. TL13). OAS-TL plays a key role in the synthesis of cysteine and glutathione, which are required for the regulation of plant responses to oxidative stress (Youssefian et al., 2001). To synthesize more sulfur-containing amino acids (cysteine and methionine), the expression levels of SAT and OAS-TL were downregulated, whereas methionine synthase expression was upregulated (Figure 7A). Methionine is involved in sulfur-adenosyl methionine synthesis, which may potentially serve as a bridge in partially facilitating sulfur's effects on grain yield and gluten components (Yu et al., 2021).

The metabolomic analysis revealed that glutathione contents of MH13 and MH18 increased by 11.9-fold and 2.7-fold compared to those under the no-sulfur treatment, respectively (Table 1). Several DEGs involved in the glutathione degradation process were observed, including gamma-glutamylcyclotransferase (4.3.2.9) (Leustek et al., 2000; Ohkama-Ohtsu et al., 2008) and glutathione S-transferase (GST, 2.5.1.18) (Figure 7B). Among them, five DEGs functionally annotated as GSTs [upregulated: *TraesCS1B02G269100* (3.1-fold), *TraesCS1D02G191100* (2.1-fold), *TraesCS3D02G261100* (3.7-fold), and *Novel.18508* (5.8-fold); downregulated: *TraesCS5D02G440700* (-1.5-fold)] significantly responded to the sulfur treatments (TH13 vs. TL13). Meanwhile, a gene (*TraesCS6A02G068300*) encoding gamma-glutamylcyclotransferase exhibited high sulfur-induced expression (TH8 vs. TL8; TH13 vs. TL13). We detected some significant enrichment of L-amino acids, namely, N6-acetyl-L-lysine (2.3-fold), L-tyrosine (2.3-fold), L-histidine (1.8-fold), L-phenylalanine (1.7-fold), and S-adenosyl L-homocysteine (0.6-fold) (Supplementary Figure S11). Furthermore, the contents of amino acids in the S60 plants were found to be significantly higher than those in the S0 plants at the three-grain developmental stages (Figure 1D). Thus, these DEGs and DEMs are considered “core DEGs and metabolites” and might be necessary for grain protein synthesis.

### Sulfur effect on starch and sucrose metabolism

The metabolomic analysis revealed that the contents of sucrose, raffinose, and GDP-alpha-D-glucose in MH13 accumulated 1.5-, 1.7- and 1.9-fold, respectively, compared to those in the no sulfur treatment (ML13), suggesting that sulfur application could promote carbohydrate formation and provide an energy source for grain filling. Sucrose and glucose are important substrates for starch synthesis, whose synthesis and degradation are mainly achieved through starch and sucrose metabolism. We also detected extensive DEG changes in this pathway, with induced starch synthesis by ADP-glucose pyrophosphorylase (AGPase) and starch branching enzyme (SBE) (Figure 7C). A gene annotated as AGPase (*novel.17599*) was observed to be significantly upregulated by the sulfur application (TH13 vs. TL13) in the RNA-seq data, suggesting that it might enhance the sink strength of developing seeds and improve the starch content (Li et al., 2011). Furthermore, a gene (*TraesCS7A02G549100*) annotated as SBE showed a higher expression level at TH13 than at TL13; this gene was the only enzyme acting on glucan to produce branches (Sawada et al., 2018). Our data confirmed its crucial role in the biosynthesis of amylopectin (Kim et al., 1998; Blauth et al., 2002). Thus, these two DEGs (*Novel.17599* and *TraesCS7A02G549100*) might play a significant role in promoting the accumulation of starch in wheat grains. Our integrated analysis of the transcriptomic and metabolic data provided evidence that the starch and sucrose metabolism pathways played a prominent role in the response to the sulfur application.

## Discussion

### Sulfur application has positive effects on both grain yield and protein content

In the current study, we observed the positive impacts of sulfur fertilization on grain yield traits and grain protein content-related traits in bread wheat. Sulfur application on the anthesis date significantly increased the grain plumpness and then led to an increase in TKW. Our results show that the sulfur application had the potential to increase grain production per plant and seed size. Moreover, starch levels in seeds are highly associated with grain yield (Gao Y. et al., 2021). The sulfur application might promote the synthesis of starch in grains, which was beneficial for increasing the grain yield. The analysis of the protein parameters showed that the S60 treatment could substantially improve GPC and grain amino acid contents. Recently, sulfur supply was reported to strongly increase the abundance of several proteins involved in glutathione metabolism (Bonnot et al., 2017). Through the analysis of the RNA-seq data, the key genes/enzymes (such as AGPase, SBE, and GSTs) involved in starch and protein synthesis in response to sulfur treatments were identified. Metabolites, such as glutathione, carbohydrates, and free amino acids, affected the responses of wheat to the sulfur application. In particular, the cysteine and methionine, glutathione, starch, and sucrose metabolism pathways were heavily involved in the grain developmental stage in this study. More in-depth studies are needed to determine how sulfur coordinates the balance between grain yield and quality.

### Sulfur application activated the glutathione metabolism pathway

Sulfur fertilization could significantly improve the glutathione content, which is important for the synthesis of sulfur-containing proteins in wheat grain (Steinfurth et al., 2012). Our data revealed that sulfur application at the anthesis stage induced a significant change in the abundance of glutathione expression in developing grains. In Arabidopsis seedlings, the contents of cysteine and glutathione were reduced upon sulfate starvation and were significantly increased after the resupply of sulfate (Bielecka et al., 2014). Sulfur deficiency caused a decrease in sucrose, glutathione, and glutathione disulfide in filling wheat grain (Dai et al., 2015). In our study, the relative level of glutathione in MH13 and MH18 was higher than that in their corresponding controls, suggesting that sulfur application can activate the glutathione metabolism pathway to accumulate more glutathione to remove active oxygen and heterologous harmful substances (Maruyama-Nakashita, 2017). The most relevant enzymes involved in glutathione metabolism are glutathione reductase, GST, glutathione hydrolase, and glutaredoxins (Takahashi et al., 2011). Two GSTs (*TmLoc013296* and *TmLoc035976*) were highly upregulated in response to sulfur deficiency (Bonnot et al., 2020). Our data highlight that four GST-encoded genes were significantly upregulated in response to the sulfur application, suggesting that GSTs might play an important role in grain development. In addition, a gamma-glutamylcyclotransferase DEG was detected in the glutathione metabolism pathway, but knowledge regarding its function in plants is limited (Leustek et al., 2000). We infer that this DEG might eventually trigger the formation of downstream amino acids since some L-amino acids were significantly enriched. Consequently, all DEGs lead to the degradation of glutathione, which may be used to provide cysteine for protein synthesis and as a precursor for numerous metabolites, thus affecting the formation of free amino acids (Ohkama-Ohtsu et al., 2008). Glutathione was significantly increased in the grain filling stages, indicating that the sulfur application was beneficial for inducing the formation of glutathione, which facilitated the formation of sulfur-containing proteins in later grain development and eventually influenced the rheological properties of dough and baking performance (Reinbold et al., 2008).

### Effect of sulfur on gene expression involved in starch and sucrose metabolism

Previous studies have reported that AGPase, granule-bound starch synthase (GBSS), soluble starch synthase, and SBE are key enzymes in starch biosynthesis (Seung and Smith, 2019). In our study, the relative level of SBE (*TraesCS7A02G549100*) was high at the early phase of seed formation (8 DPA) and slightly increased at 13 DPA when starch synthesis occurred in the endosperm. However, the DEGs mentioned above (*TraesCS7A02G549100* and *novel.17599*) remained unchanged between TH18 and TL18. These expression patterns imply that these DEGs could regulate the formation of grain starch in the early stage of grain development of GY2018. Amylose in the cereal endosperm is synthesized by GBSS (Pfister and Zeeman, 2016). However, no significant DEGs were detected in genes encoding GBSS, a key enzyme for amylose synthesis; thus, we hypothesized that sulfur application might primarily promote the synthesis of amylopectin rather than amylose. In this study, the S60 application at anthesis increased the contents of sucrose and glutathione in developing wheat grains. The accumulation of sucrose might stimulate the expression of starch-related genes, thereby promoting the accumulation of starch and resulting in a higher seed yield and larger grain size (Smidansky et al., 2003).

### Sulfur supplied on anthesis could compensate for sulfur deficiency in grain

Our data emphasize the importance of understanding the timing of regulatory events in response to sulfur supply. Most DEGs and DEMs in response to the sulfur application were significantly accumulated at 13 DPA, indicating that the grains were able to maintain amino acid homeostasis at the early stages of grain development. Recent studies have shown that sulfur fertilization at the ear emergence stage could prevent sulfur deficiency at later stages (Steinfurth et al., 2012), and its supply at 490°C days (thermal time: growing degree days) after anthesis could also efficiently mitigate sulfur deficiency, leading to a rapid recovery in sulfur-rich grain storage proteins and some metabolites (e.g., glutathione, citrate and free amino acids) (Dai et al., 2015). In this study, our data show that the S60 application at anthesis could prevent sulfur deficiency in the late stages of wheat growth. The sulfur application at anthesis significantly increased the contents of several metabolites (e.g., glutathione, sucrose, and free amino acids) in grains. Our data pinpointed a critical stage in the grain development response to deficiency, which could provide guidance for the application of sulfur fertilizers in field production.

### Key stages of DEGs and DEMs

We integrated our transcriptomic and metabolomic data obtained from 8 to 18 DPA to analyze the effects of the sulfur treatments on developing grains. It is known that wheat grain development basically reaches morphogenesis at 13 DPA, and then, the grain begins to accept the transformation of nutrients into the grain, forming starch and accelerating grain filling. The changing trend of glutamine synthetase activity and ABA content in grains of the two varieties maintained a high value at 10 and 15 DPA, respectively, and then sharply decreased (Zhu, 2008). The results show that the application of sulfur fertilizers could improve the ability of nitrogen assimilation in wheat grains to meet the needs of physiological metabolism. The peak of IAA content in the sulfur treatment group appeared before the peak of grain filling, suggesting that it was beneficial for the differentiation and proliferation of endosperm cells and promoted the input of more assimilates. Previous results showed that chloroplasts completely disappeared at 15 DPA. Meanwhile, the surface of starch granules possessed deep electron staining; it may be some functional protein for amylolysis (Zhou et al., 2009). In our study, the abundance of sucrose and glucose accumulated significantly at 13 DPA, and the significantly enriched GO term was protein synthesis, suggesting that the increased sucrose uptake into the grain should increase the sink strength and subsequently promote seed protein synthesis. Programmed senescence may have occurred between 8 and 16 DPA, and accelerated senescence is beneficial for increasing the grain protein content (Gregersen et al., 2008). Our data show that most DEGs and DEMs were found at 13 DPA when compared with 8 and 18 DPA, suggesting that the life activity was exuberant during this period, which resulted in a lack of separation at 8 and 18 DPA.

### Transcription factors in response to sulfur treatments

In plants, MYB represents one of the largest TF families (Riechmann et al., 2000). Specifically, MYB28, MYB29, and MYB76 can regulate lipid glucosinolates, which are derived from methionine (Hirai et al., 2007). It has been reported that these three MYBs activate the sulfate reduction pathway, which is required for glucosinolate production (Yatusevich et al., 2010). Plants assimilate inorganic nitrate into cysteine and other sulfur-containing primary metabolites to synthesize secondary metabolites, e.g., glucosinolate (Mugford et al., 2011). In this study, *TraesCS3B02G400500* might play a positive regulatory role in glucosinolate synthesis due to its differential expression under the sulfur treatments and homology with *AtMYB118*. However, although *TraesCS2D02G324800, TraesCS2B02G343800*, and *TraesCS2A02G338200* were homologous to *AtMYB29* (Stracke et al., 2001, alignment rate >74%), their expression was not affected under the sulfur treatments. Here, several MYB TFs were identified to be involved in glucosinolate synthesis pathways (Li L. et al., 2021). However, as described above, some genes were not differentially expressed, showing the complexity of MYBs involved in sulfur metabolism and indicating that homologous genes in different species may have different functions. How MYB TFs play a role in the sulfur-mediated regulatory network responsible for grain quality and yield formation in wheat requires subsequent experimental verification.

### Sulfur application could regulate grain protein synthesis through different pathways

This study revealed a sulfur-mediated regulatory mechanism responsible for grain protein biosynthesis, which was verified through integrative transcriptomic and metabolomic assays. Under sulfur deficiency, changes in the grain storage protein composition could be controlled by the pool of total free amino acids, the grain nitrogen-to-sulfur ratio, and sulfur deficiency-responsive genes (Bonnot et al., 2020). The sulfur application could increase the nitrogen use efficiency by regulating glutamine synthetase activity, thus facilitating the biosynthesis of aspartate-family amino acids to synthesize more glutamic acid and ultimately improving the processing quality (Yu et al., 2021). The effect of sulfur application on grain quality is a complex process. In this study, in total, 542 DEMs were revealed, including 69 amino acid metabolites (Supplementary Figures S9A,B), most of which were upregulated in response to the sulfur application. The significantly upregulated differential metabolites included L-cystine (1.6-fold) and D-methionine (1.9-fold) compared to no sulfur supply (ML8), which are important substrates for sulfur-containing protein synthesis and play vital roles in human health and nutrition (Ingenbleek and Kimura, 2013). However, the expression abundance of D-methionine was significantly decreased in MH13 and MH18 compared with that in their corresponding controls, indicating that sulfur might promote the synthesis of more methionine into downstream metabolites. Sulfur may promote the formation of sulfur-containing amino acids in the early grain developmental stage, and these amino acids contributed to the synthesis of sulfur-containing proteins in later grain development. Therefore, a sufficient supply of sulfur is an important procedure in wheat crop management to improve the end-product quality and enhance the health benefits for consumers.

## Conclusion

In this study, the S60 treatment in GY2018 resulted in 13.2% and 3.6% increases in grain yield and grain protein content, respectively. Global dynamic changes in the transcript and metabolic profiles were comprehensively analyzed in the wheat variety GY2018, which showed a positive response to the sulfur application at both the transcriptional and metabolic levels. Overall, 10,694 DEGs and 542 DEMs were detected, including 69 amino acid metabolites. The combined transcriptomic and metabolomic analyses suggested that the sulfur sensitivity of GY2018 may be related to increased glutathione levels and the activation of the glutathione metabolism pathway; enhanced amino acid metabolism and the accumulation of some sulfur-related amino acids; promoted sucrose accumulation, providing energy for starch accumulation at later stages of grain development and activating the starch and sugar metabolic pathway; and identified a TF (MYB118) that may play an important role in the sulfur metabolism process by regulating the synthesis of glucosinolate. Further functional evaluation of sulfur-responsive DEGs or metabolic pathways will facilitate the understanding of starch and protein synthesis in bread wheat, and these candidate genes and their involved networks could contribute to breeding new varieties with high yield potential and protein quality and improve wheat production through sulfur cultivation.

## Data availability statement

The original contributions presented in the study are publicly available. This data can be found at: https://ngdc.cncb.ac.cn/gsa/, CRA006056.

## Author contributions

RL conceived and designed the experiments. DL analyzed the data, reviewed, and edited the manuscript. ZL, XF, and SL performed the experiments. ZL, XD, YZ, and WZ analyzed the format. HY contributed materials. SH provides technical guidance on variety cultivation. ZL and RL wrote the manuscript. All authors read and approved the final version of the manuscript.

## Funding

This research was supported by the National Key Research and Development Program of China (2017YFD0300909) and the China Agriculture Research System (CARS-03-05).

## Conflict of interest

The authors declare that the research was conducted in the absence of any commercial or financial relationships that could be construed as a potential conflict of interest.

## Publisher's note

All claims expressed in this article are solely those of the authors and do not necessarily represent those of their affiliated organizations, or those of the publisher, the editors and the reviewers. Any product that may be evaluated in this article, or claim that may be made by its manufacturer, is not guaranteed or endorsed by the publisher.
