# Supplementary material for: Integrated transcriptomic and metabolomic analyses revealed the regulatory mechanism of sulfur application in grain yield and protein content in wheat (Triticum aestivum L.)
Source: Front Plant Sci. 2022 Sep 16;13:935516. doi: 10.3389/fpls.2022.935516 (PMC9523790; doi:10.3389/fpls.2022.935516)
Supplement: Supplementary file 1 [file Data_Sheet_1.docx]

Supplementary Material

# Supplementary Figures and Tables

## Supplementary Figures


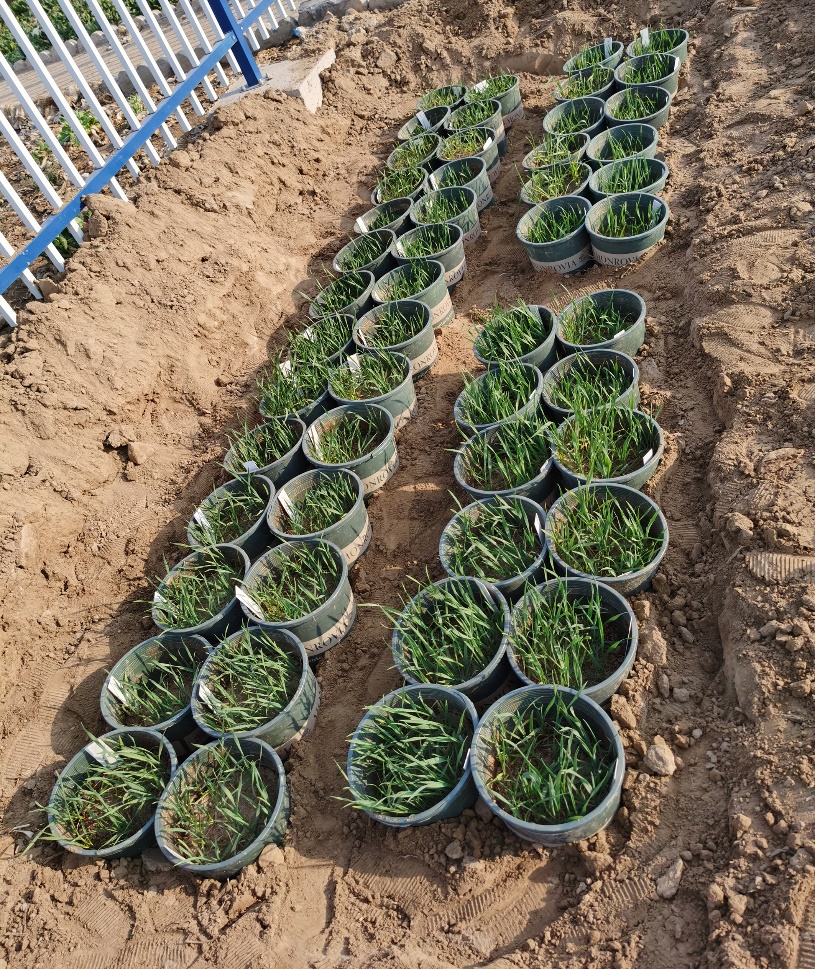


**Figure S1.** Photograph of a plant grown in the fields. The size of the polyethylene pot was 24 cm × 24 cm × 27 cm.


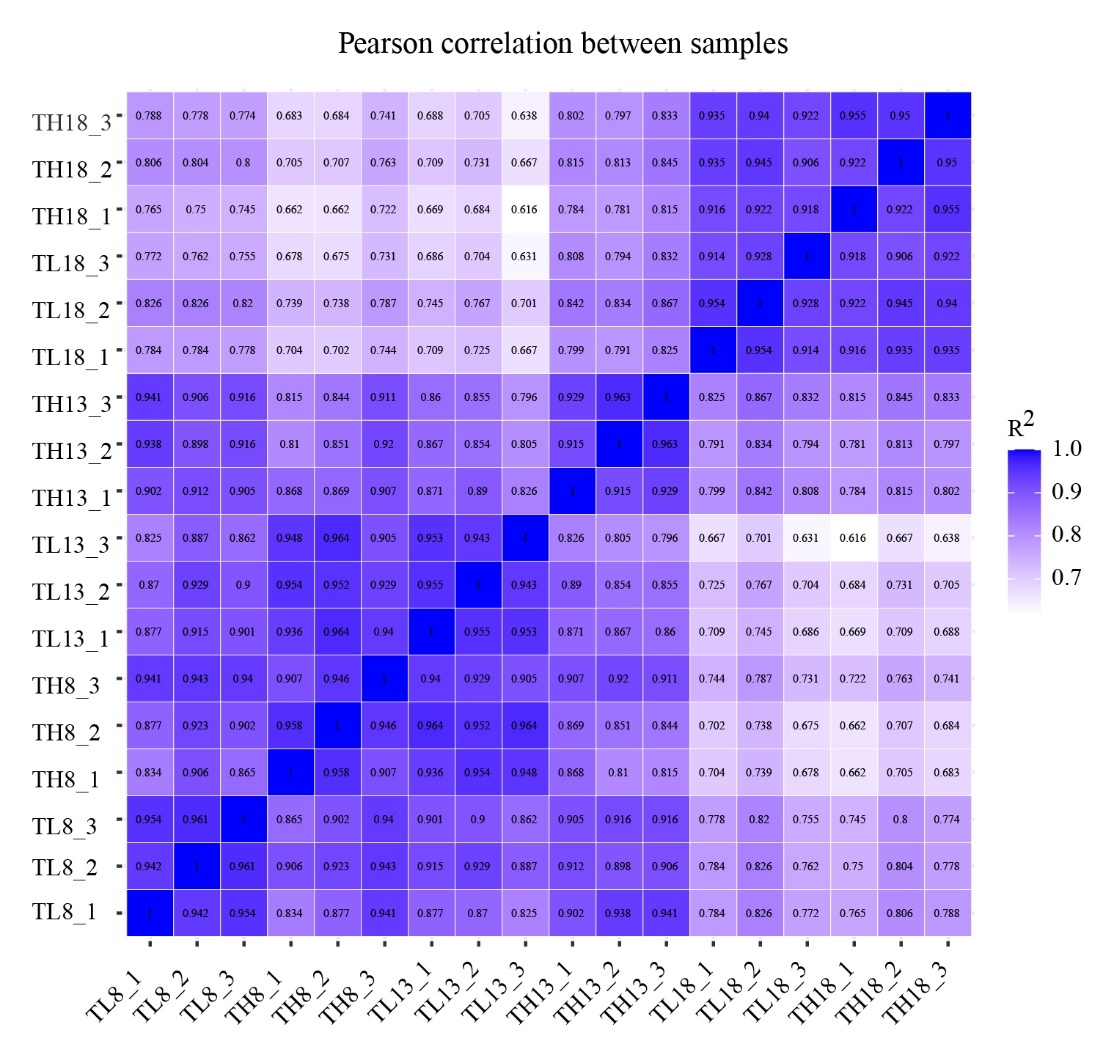


**Figure S2.** RNA sequencing outcomes of the wheat variety GY2018 under the S60 and S0 treatments. Heatmap representing the Pearson rank correlation coefficients between pairs of samples based on the global expression profiles under the S60 and S0 treatments at 8, 13 and 18 DPA.


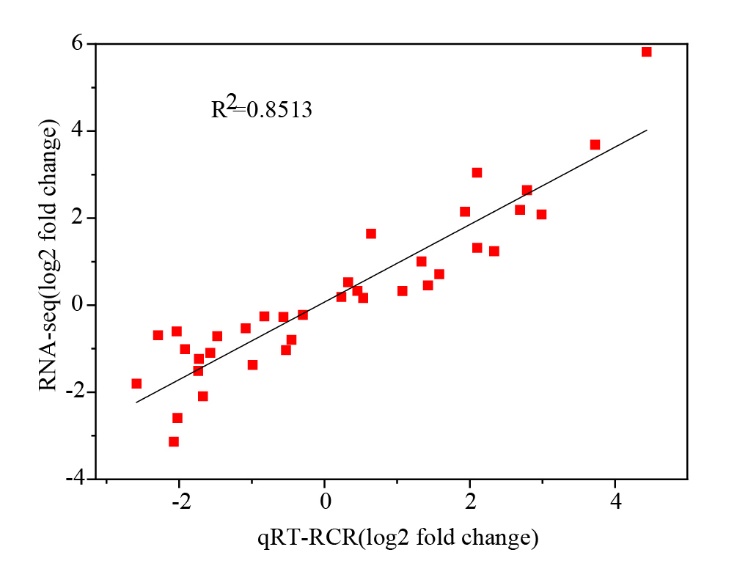


**Figure S3.** Correlation analysis between the RNA-seq and qRT‒PCR data (Pearson’s correlation analysis). Log2 (fold change) values of qRT‒PCR data (x-axis) are plotted against log2 (fold change) values of RNA-seq data (y-axis).


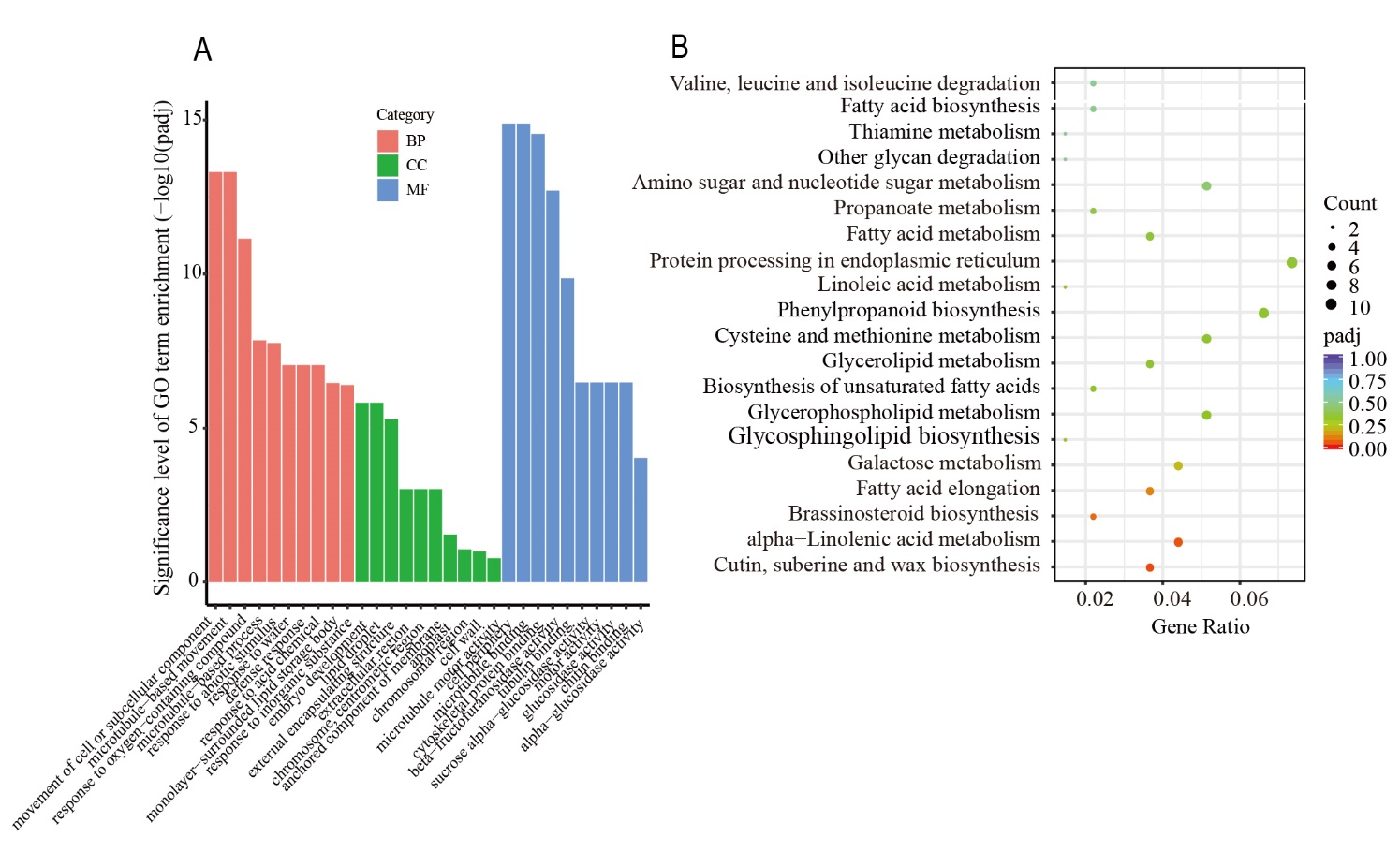


**Figure S4.** GO classification and KEGG enrichment of the DEGs in the TH8 vs. TL8 comparison groups of GY2018. (A) Significantly enriched GO terms (corrected *P value* ≤ 0.05). The y-axis represents the significance level of GO term enrichment, and the x-axis represents each GO term. Biological process, red bar; Cellular component, green bar; Molecular function, blue bar. (B) KEGG pathway enrichment scatter diagram of the DEGs. The colours of the dots indicate the correlated *P value*, and the sizes of the dots indicate the input number. Abbreviations: DPA - days post-anthesis, DEGs - differentially expressed genes.


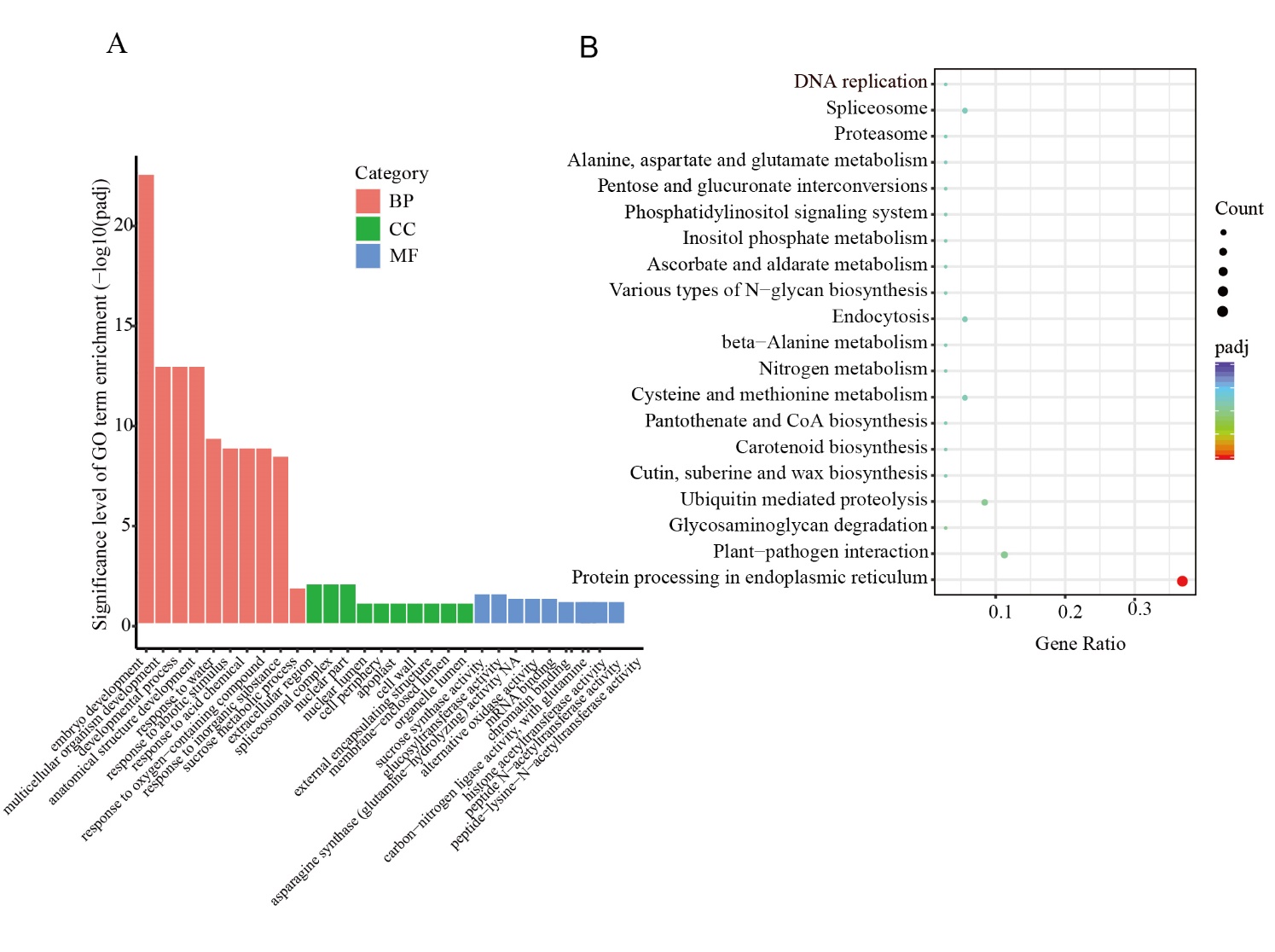


**Figure S5.** GO classification and KEGG enrichment of the DEGs in the TH18 vs. TL18 comparison groups of GY2018. (A) Significantly enriched GO terms (corrected *P value* ≤ 0.05). The y-axis represents the significance level of GO term enrichment, and the x-axis represents each GO term. Biological process, red bar; Cellular component, green bar; Molecular function, blue bar. (B) KEGG pathway enrichment scatter diagram of the DEGs. The colours of the dots indicate the correlated P value, and the sizes of the dots indicate the input number. Abbreviations: DPA - days post-anthesis, DEGs - differentially expressed genes.


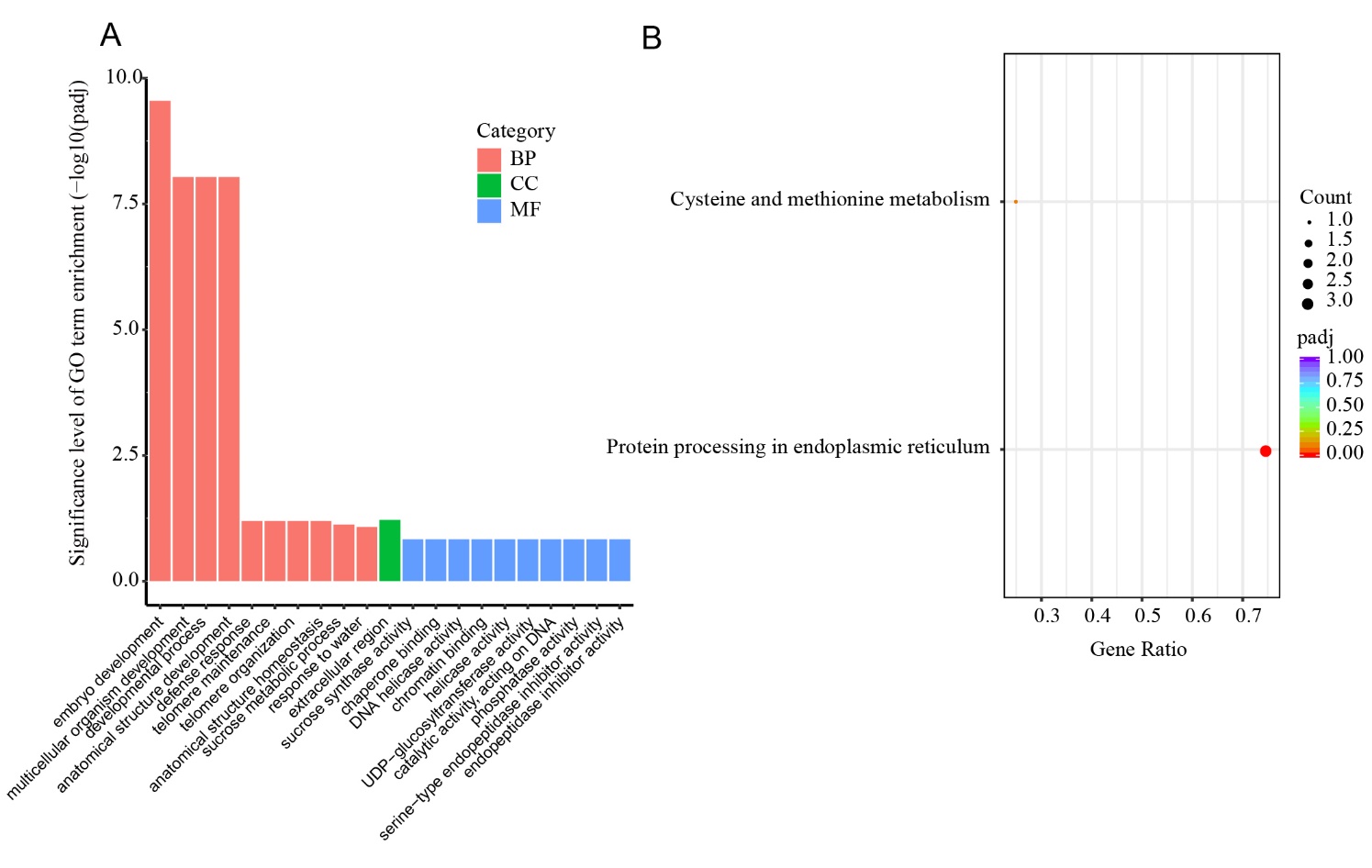


**Figure S6.** GO classification and KEGG enrichment of 101 common DEGs in all three comparison groups of GY2018. (A) Significantly enriched GO terms (corrected *P value* ≤ 0.05). The y-axis represents the significance level of GO term enrichment, and the x-axis represents each GO term. Biological process, red bar; Cellular component, green bar; Molecular function, blue bar. (B) KEGG pathway enrichment scatter diagram of the DEGs. The colours of the dots indicate the correlated P value, and the sizes of the dots indicate the input number. Abbreviations: DPA - days post-anthesis, DEGs - differentially expressed genes.


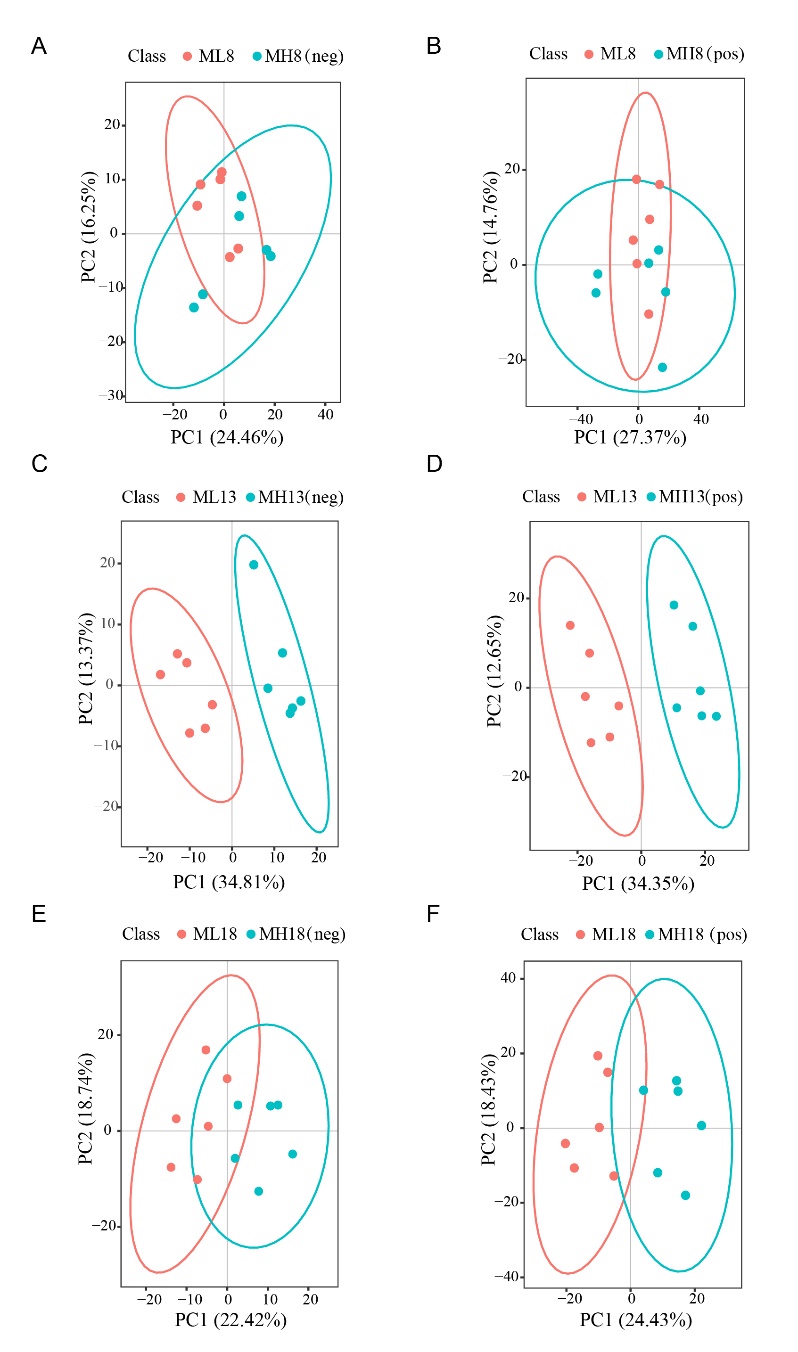


**FIGURE S7.** Pairwise principal component analysis of DEMs between the S60 and S0 groups at each time point. (A), (C), (E) Presentation of the DEMs under the S60 and S0 treatments in the negative (neg) mode. (B), (D), (F) Presentation of the DEMs under the S60 and S0 treatments in the positive (pos) mode.


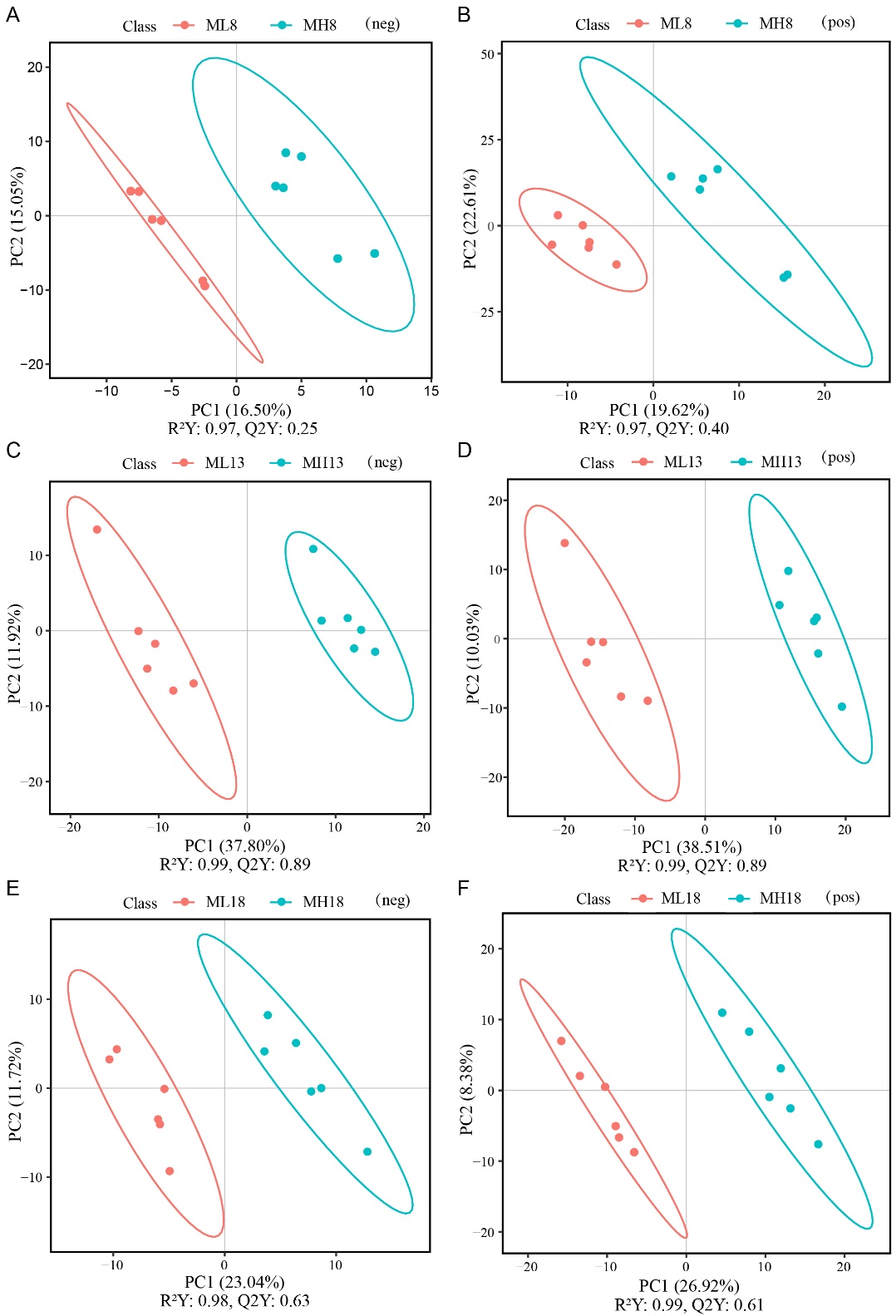


**Figure S8.** Partial least squares discriminant analysis score chart of the DEMs between intergroup differences and intragroup repeatability. (A), (C), (E) Presentation of the DEMs under the S60 and S0 treatments in the negative (neg) mode. (B), (D), (F) Presentation of the DEMs under the S60 and S0 treatments in the positive (pos) mode.


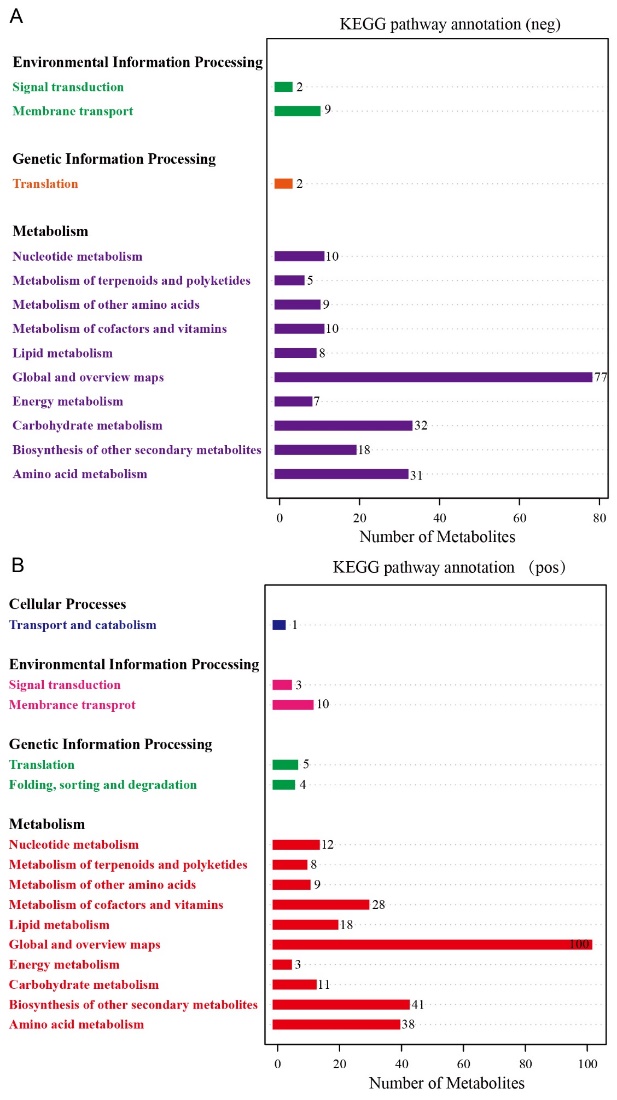


**Figure S9.** Analysis of the differentially expressed metabolites (DEMs) based on the KEGG pathway database of two ion modes in the MH8 vs. ML8, MH13 vs. ML13 and MH18 vs. ML18 comparison groups of GY2018. (A) Presentation of the DEMs under the S60 and S0 treatments in the negative (neg) mode. (B) Presentation of the DEMs under the S60 and S0 treatments in the positive (pos) mode.


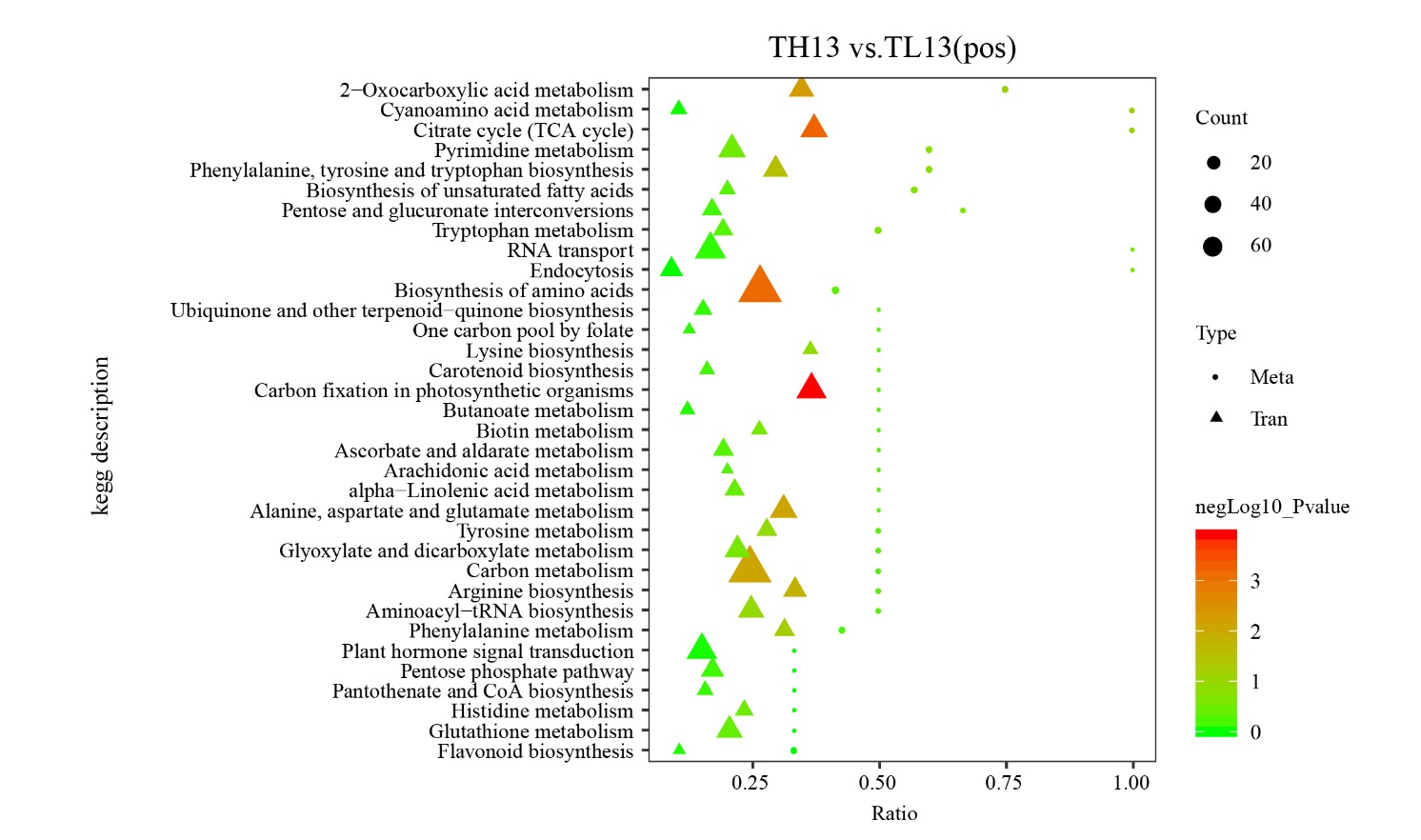


**Figure S10.** KEGG pathway of the coenrichment of DEGs and DEMs at 13 DPA according to a joint analysis of transcription and metabolism. The x-axis (Ratio) represents the ratio of differential metabolites or genes enriched in this pathway to the number of metabolites or genes annotated in this pathway, and the y-axis represents the KEGG pathway enriched by metabolites-transcriptome. Count represents the number of metabolites or genes enriched in the pathway.


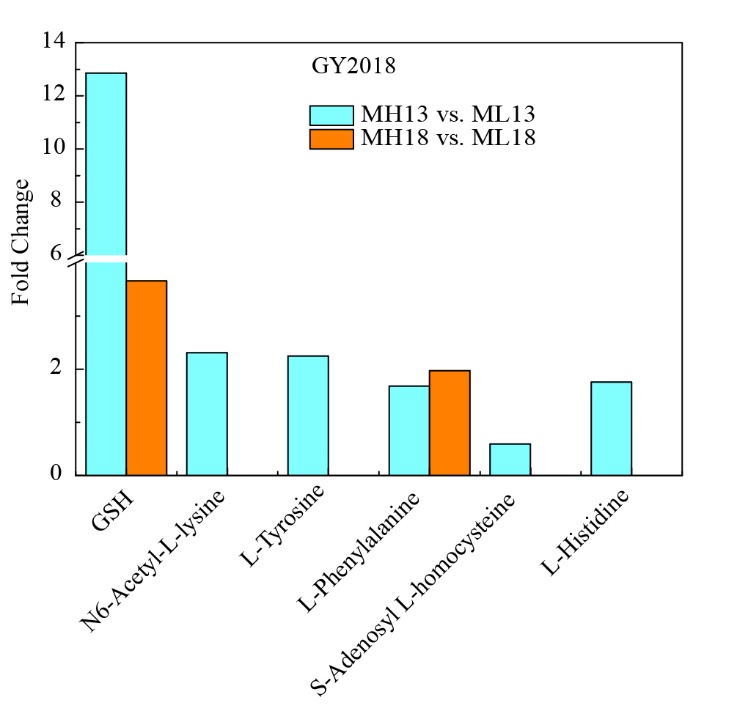


**Figure S11.** Differences in the metabolites involved in the glutathione metabolism pathway. Different coloured columns represent two comparison groups. The labels are ML13 (13 DPA, S0), MH13 (13 DPA, S60), ML18 (18 DPA, S0), and MH18 (18 DPA, S60).
